# Intermittent fasting and mental and physical fatigue in obese and non-obese rats

**DOI:** 10.1371/journal.pone.0275684

**Published:** 2022-11-02

**Authors:** Paige Niepoetter, Carolyn Butts-Wilmsmeyer, Chaya Gopalan

**Affiliations:** 1 Department of Biological Sciences, Southern Illinois University Edwardsville, Edwardsville, IL, United States of America; 2 Center for Predictive Analytics, Southern Illinois University Edwardsville, Edwardsville, IL, United States of America; 3 Department of Applied Health, Southern Illinois University Edwardsville, Edwardsville, IL, United States of America; 4 Department of Nurse Anesthesiology, Southern Illinois University Edwardsville, Edwardsville, IL, United States of America; University of Lübeck: Universitat zu Lubeck, GERMANY

## Abstract

Intermittent fasting (IF) is an alternating pattern of restricting eating. This study evaluated mental and physical fatigue secondary to IF (daily 18-hour fast, 7-days-a-week) in the high-fat diet (HFD)-induced male obese Sprague Dawley rats. Fifty-four rats were randomly assigned to a HFD (n = 28) or a standard diet (SD; n = 26). After six weeks, the HFD rats were divided into one of four groups: obese HFD *ad libitum* (OB-HFD-AL), obese HFD-IF (OB-HFD-IF), obese SD-AL (OB-SD-AL), and obese SD-IF (OB-SD-IF). Similarly, non-obese controls were grouped into HFD-AL (C-HFD-AL), non-obese HFD-IF (C-HFD-IF), non-obese SD-AL (C-SD-AL), and non-obese SD-IF (C-SD-IF). After 2 weeks of IF, mental and physical fatigue were measured using open field (OF) and novel object recognition (NOR) tests. Rats on IF gained weight at a slower pace (*p<*0.05) and had lower glucose levels (*p*<0.01) compared to the AL group. In non-obese rats, ketone levels were higher in the IF-HFD group than IF-SD (p<0.05) and AL-SD (*p*<0.01) animals. Obese rats exhibited elevated blood ketone levels in IF-SD conditions versus AL-SD rats (*p*<0.01). AL-HFD rats had higher ketone levels than AL-SD animals in both obese and non-obese groups (*p*<0.05). In conclusion, rats with higher blood ketone levels, whether they were on IF or AL, traveled a greater distance during OF suggesting a lack of physical fatigue. There was no significant difference between IF and AL during NOR indicating a lack of mental fatigue. Thus, IF results in reduced body weight and blood glucose levels but does not induce physical or mental fatigue.

## Introduction

Obesity is associated with cardiovascular disease, type 2 diabetes, several types of cancers, mental illnesses, cognition impairment, and chronic neurological degenerative conditions such as Alzheimer’s disease and dementia [1–3]. For example, Simon et al. reported that obesity is associated with an approximately 25% increase in mood and anxiety disorders [4]. A meta-analysis of 17 studies found that individuals with obesity were 1.26 times more likely to experience depression compared to non-obese individuals [2]. Multiple lifestyle modifications have been studied to combat the adverse effects of obesity, including dietary measures such as intermittent fasting (IF) [5, 6].

IF has been practiced for many decades in several different religions. Followers of Islam engage in IF during the holy month of Ramadan, where fasting takes place every day of the month ranging between 11–22 hour intervals [7]. While Ramadan fasting is one example of time-restricted feeding, other IF regimens include alternate-day fasting switching between consuming no calories and regular food intake every other day. Some follow modified fasting regimens that consume 20% of the average daily caloric intake on fasting days, such as in the case with the popular 5:2 diet, where fasting occurs 2 days a week nonconsecutively, with the other five days consisting of regular food intake. IF, irrespective of the strategy used, is shown to produce beneficial effects, including increased insulin sensitivity, weight loss, and reductions in plasma cholesterol levels [7–9].

The IF regimen is expected to induce certain cellular changes such as depleted levels of glycogen stores and increased blood ketones as a result of higher rate of lipolysis [10, 11]. Weight loss and baseline blood glucose levels are often the byproducts of increased energy expenditure through lipid metabolism [10, 12]. The beneficial effects of IF are demonstrated in a study by Spezani et al. (2020). Twelve-week-old C57BL/6J mice were fed either a control diet (C; 10% kcal fat), a HFD (50% kcal fat), or a high fructose diet (HFru; 50% kcal fructose) for eight weeks. After these eight weeks, half of the rats started on an alternate day IF regimen (IF; 24 h fed, 24 h fasting) while still maintaining their diet type for four weeks. All groups tested benefited from IF with improved glycemic control, reduced insulin resistance, and weight loss [13]. These findings were reiterated by Gotthardt and Bello (2017) using alternate day IF (IMF) in adult obese male C57BL/6 mice [14].

Obesity is linked to low endurance and increased fatigue in humans [15, 16]. IF may pose a solution to obesity-related fatigue, though assessments of fatigue on this regimen are scarce and the results are often mixed [5, 17, 18]. Self-assessments of fatigue in those participating in Ramadan fasting have reported increased fatigue while on the regimen. A study by Chaouachi et al. (2009) with male judo athletes, during Ramadan, reported increased fatigue, though physical performance was relatively unchanged [18]. A group of nurses completed a similar fatigue self-assessment and reported increased fatigue while fasting, with fatigue increasing as subjective health scores declined [19]. Other studies have shown improvement in subject fatigue scores during times of IF. A study by Bowen et al. (2018) used self-assessments to measure fatigue in obese individuals partaking in a high-protein diet with or without alternate-day fasting. They reported that fatigue decreased overall, but those also participating in alternate-day fasting had even greater levels of improvement [17]. A meta-analysis performed by Abadia, Daab, and Bouzid related physical measures to Ramadan fasting and found that power and sprinting measures were reduced after Ramadan, though aerobic performance fatigue index scores were not influenced [20]. Overall, such mixed results produced by human studies related to IF and fatigue demonstrate the knowledge deficit that must be filled in order to positively state that IF can mitigate the fatigue associated with obesity. It has been suggested that the IF regimen may mitigate physical fatigue by providing another fuel source in ketones such as beta-hydroxybutyrate (BHB), which can be used once carbohydrates have been depleted [21, 22]. Moreover, elevated ketone levels are associated with greater time to fatigue in rodents exposed to forced swim tests and forced walking models [23, 24], though additional literature in rodent models is scarce.

Obesity has also been associated with impairments in cognition, the higher-order process of learning, memory formation, and retrieving information through thought, experience, and the senses [4, 25–27]. Considering the many benefits of IF, some studies have investigated its potential for improving cognitive functions as well, though this information is limited [8, 28, 29]. A study by Li et al. (2013) exposed 7-week-old mice to control, HFD, or alternate-day fasting with SD conditions over 11 months. After this time, the mice underwent a Barnes maze test, which measures spatial working memory (SWM) by recording the amount of time it takes to enter the correct target box that was previously introduced during a habituation phase. The mice in the fasting condition exhibited better memory and cognition during the Barnes maze test than mice in the other conditions [30]. Many studies attribute this improvement in cognition to caloric restriction as the window of food intake is limited [31]. Geng et al. (2007) investigated the cognitive effects of a 60% calorie-restrictive diet in 18-month-old versus *ad libitum* rats over six months. At the end of this study, a Morris water maze test was performed, where SWM was measured by how long it took for a rat to find an escape platform that they were previously introduced to and found that rats on the calorie-restrictive diet outperformed the *ad libitum* group in the Morris water maze test [32]. These studies suggest that caloric restriction via IF is responsible for the beneficial cognitive effects experienced, though it is likely that deeper cellular mechanisms are at play. For example, elevated ketone levels may provide alternative energy sources for cognitive functions [33]. A study by Murray et al. (2016) found improved memory during radial arm maze testing in rats fed a high ketone ester diet which resulted in higher plasma beta-hydroxybutyrate levels after 36 days of this dietary intervention [24]. Our previous study using HFD-induced obese rats had increased ketone levels which were associated with greater time spent with novel versus familiar objects in NOR testing, indicating that ketones protected against memory deficits [34]. Ketones may exert these benefits in a number of ways. It is thought that the neuroprotective qualities of ketones stem from increasing energy production by promoting mitochondrial reproduction in neurons and reducing neuronal apoptosis [22, 35]. It is also possible that the metabolic switch from glucose to ketone utilization could increase the expression of brain-derived neurotrophic factor (BDNF), which has been shown to improve cognition by increasing neurogenesis, synaptogenesis, and preventing apoptosis [36–38].

Since the connection between IF and fatigue is not well understood in rodents and is often subjective in humans, additional exploration is needed to better understand the benefits IF has to offer, as well as potential disadvantages [5]. The goal of this study was to utilize behavioral testing after exposure to IF in obese and non-obese rats fed either a SD or HFD in the hopes of gaining a better understanding of the connection between ketones and mental and physical fatigue. It was hypothesized that IF would protect against mental and physical fatigue via increased ketone (BHB) levels [39].

## Materials and methods

### Animals

Fifty-four male Sprague Dawley rats at 7 weeks of age were received from Envigo Labs, Indianapolis, IN. They were housed individually under controlled laboratory conditions (12-hour light/dark cycle with lights on at 7:00 PM at a room temperature of 20.0–22.2°C) in solid-bottom cages with aspen chip bedding. All protocols described were approved by the Southern Illinois University Edwardsville Institutional Animal Care and Use Committee (040618-CG2).

### Diet

Upon arrival, animals were randomly placed into one of the 2 diet groups. The first 6 weeks of the study consisted of inducing obesity by feeding one group of rats a HFD (n = 28; formula D12492 from *Research Diets Inc*) while the rest received a SD (n = 26; *Mazuri* rat chow 5663; Table 1).

**Table 1:** Macronutrient composition (Niepoetter et al. 2021).

	HFD	SD
Fat (kcal)	60%	17%
Carbohydrate (kcal)	20%	56%
Protein (kcal)	20%	27%
Energy Density (kcal/g)	5.21	3.41
Fat Source	lard, soybean oil	flaxseed oil, polyunsaturated fatty acids

HFD: high-fat diet; SD: standard diet; kcal: kilocalories; kcal/g: kilocalories per gram of food.

### Metabolic testing

Capillary blood sampling was used to obtain overnight fasting glucose (mg/dL) and BHB (mmol/L) levels between 7:00 and 9:00 am on day 6 each week. Blood samples for this testing were obtained by pricking the rats’ tail veins using 26-gauge lancets. Results were obtained immediately using a *Keto-Mojo* (Napa Valley, CA) glucose and ketone meter (model TD-4279). Previous studies have used capillary sampling and ketone test strips to measure circulating levels of BHB [40, 41].

### Intermittent fasting

**Fig 1:**
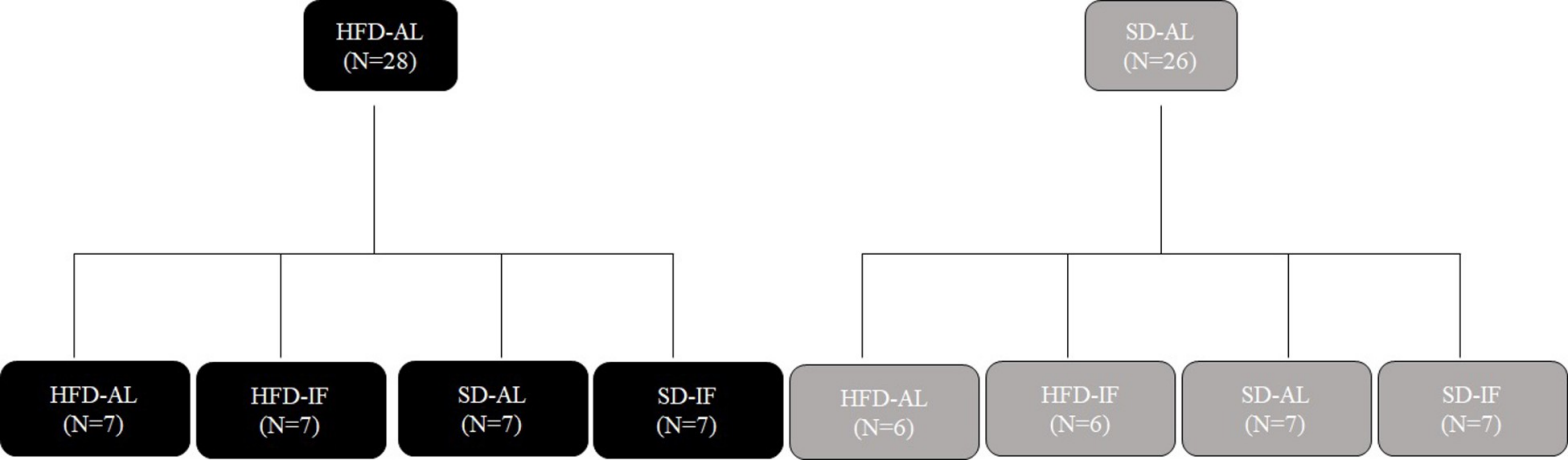
Illustrates the groups and their treatments. High-fat diet (HFD); Standard diet (SD); Intermittent fasting (IF); *Ad libitum* (AL).

Once diet-induced obesity (DIO) was achieved and behavioral tests were completed, the animals that were fed HFD were referred to as obese (OB), and those that received SD became non-obese controls (C). Both the HFD and the SD groups were divided into four subgroups each (Fig 1; Table 2): obese HFD *ad libitum* (OB-HFD-AL), obese HFD-IF (OB-HFD-IF), obese SD-AL (OB-SD-AL), obese SD-IF (OB-SD-IF), non-obese HFD*-*AL (C-HFD-AL), non-obese HFD-IF (C-HFD-IF), non-obese SD-AL (C-SD-AL), and non-obese SD-IF (C-SD-IF). Animals in the IF groups were fasted for 18 hours per day, 7 days a week. Animals were on IF for 2 weeks before behavioral testing was initiated. After 2 weeks on IF, behavioral tests were repeated to evaluate physical and mental fatigue.

**Table 2:** Study design.

Study Phase	Duration	Protocol
Induction of Obesity	Weeks 0–6	28 rats on HFD, 26 rats on SD. Blood testing and body weight measurements. OF (8 trials- one trial per day for 8 consecutive days) and NOR (at 0, 1, 3, & 7 day intervals)
Intermittent Fasting	Weeks 7–10	Rats were divided into 8 groups: OB-HFD-AL, OB-HFD-IF, OB-SD-AL, OB-SD-IF, C-HFD-AL, C-HFD-IF, C-SD-AL, C-SD-IF Blood testing and body weight measurements. OF and NOR tests as above

High-fat diet (HFD); Standard diet (SD); Obese (OB); Control (C), Open field testing (OF); Novel object recognition testing (NOR); Intermittent fasting (IF).

### Behavioral testing

Baseline behavioral testing occurred during the last 2 weeks of inducing obesity. The common definition of physical fatigue is the inability of muscles to maintain a needed level of power during and after physical activity, which is measured in a variety of ways in humans, but is commonly assessed by OF in rodents [16, 29]. During OF, movements such as the number of line crossings, distance/time moving, and motion freezing are recorded [29]. OF measures exploratory behavior and movement to indicate the physical health of rodents. The OF apparatus utilized a 100 cm x 100 cm opaque plexiglass arena with central and peripheral zones. The animal was placed into the arena to explore freely for 6 minutes. The movements were tracked using ANY-maze video tracking system (Stoelting, Wood Dale, Illinois). The total distance traveled was recorded to examine the locomotor activity of the animal. OF was performed for eight consecutive days in the dark under red light conditions during their night cycle. Mental fatigue, the impairment of cognitive performance due to the reduced mental alertness or the feeling of absence of energy, is evaluated by NOR testing in rodents [42, 43]. NOR testing measuring recognition memory provides an indirect assessment of cognition [42]. After the completion of OF testing over 8 consecutive days, the animals underwent a NOR study. Five objects of different shapes, colors, and dimensions were utilized for this study. During the familiarization phase, one object was placed in the same opaque plexiglass arena as used in OF, and the rats were then allowed 5 minutes to investigate the object within the arena. The animal was then returned to a holding cage for an inter-exposure interval (IEI) before being returned to the arena. The familiarization phase was immediately followed by the first IEI called the 0-hour test. The remaining IEIs occurred at 24-hours (one day), 72 hours (3 days), and 168-hours (7 days) after the initial 0-hour test. The duration spent by the rats investigating the new object compared to the familiar object translates to the animal’s recognition and provides a numerical measurement for memory [34, 42].

### Statistical analysis

#### A priori power analysis and data quality assurance

G*Power (version 3.1.9.4) was used to calculate the sample size needed to obtain a power of at least 0.8 at an α = 0.05. Power calculations were based on a moderate correlation among repeated measurements (r = 0.5) and a moderate effect size (η^2^ = 0.15). If rats did not gain at least 10% on the HFD relative to the mean of the SD control, these animals were excluded from the analysis. As such, the initial calculated sample size of 44 individuals was increased by a factor of 22.7% (10 individuals) to buffer against non-responders.

#### ANOVA of individual variables

Measurements of body weight, blood glucose and ketones as well as the behavioral data analysis during DIO is previously published [34]. In this study, body weight, blood glucose, and blood ketones were measured once per week and the averages of each of these variables analyzed using a repeated measures analysis of variance (ANOVA) in PROC MIXED of SAS (version 9.4). Behavioral measurements (i.e., total distance traveled and time spent with novel versus familiar objects) were measured on a daily timescale after 2 weeks of IF treatment. Specifically, distance traveled was measured daily for 8 days and the variables in NOR testing were collected at days 31 (0), 32 (1), 34 (3), and 38 (7). As such, a repeated measures ANOVA was also used, but the frequency of the repeated measurement differed between these and the weekly model. Additionally, the novel preference was first calculated with the total amount of time spent with the novel object, then as the natural log of the ratio of time spent with the novel object to the time spent with the familiar object. A correlation analysis of ketone levels and behavioral measurements during OF and NOR was performed using the cor.test function in R (version 4.0.4).

## Results

### Physiological measurements

**Fig 2:**
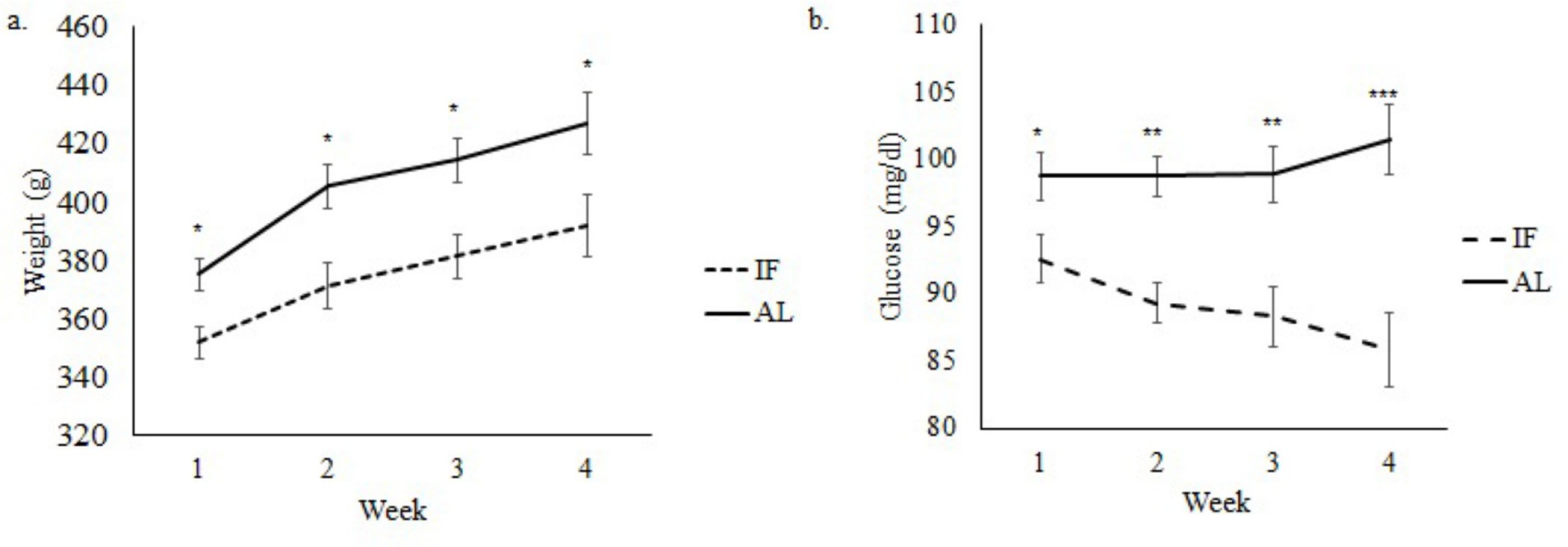
Body weight (a), and blood glucose (b) in the non-obese group. * *p*<0.05; ** *p*<0.01; ***p<0.001. IF: intermittent fasting; AL: *ad libitum*; HFD: high-fat diet; SD: standard diet. Error bars represent standard error.

**Fig 3:**
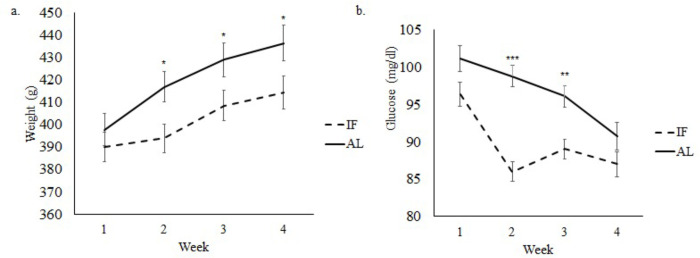
Body weight (a) and blood glucose levels (b) in the obese group.:* *p*< 0.05; **p<0.01; ***p<0.001. IF: intermittent fasting; AL: *ad libitum*; HFD: high-fat diet; SD: standard diet. Error bars represent standard error.

Both non-obese (*p*<0.01; Fig 2) and obese (*p*<0.05; Fig 3) rats on IF weighed an average of 22g less than AL rats. Diet type had a significant effect on glucose levels in both non-obese and obese rats, with HFD exhibiting higher glucose levels than SD-fed rats. Glucose levels were significantly lower in the non-obese rats undergoing IF, when compared to AL rats (*p*<0.01; Fig 2). Obese rats on IF also had lower glucose levels compared to AL rats, reaching significant levels at weeks 2 and 3 (*p*<0.01; Fig 3).

In non-obese rats, ketone levels were higher in the IF-HFD group compared to the IF-SD (p<0.05) and AL-SD (*p*<0.01) groups (Table 3). Obese rats exhibited higher blood ketone levels in IF-SD conditions versus AL-SD rats (*p*<0.01; Table 4). In both obese and non-obese groups, AL-HFD animals had higher ketone levels than AL-SD rats (*p*<0.05; Tables 3 and 4).

**Table 3:** Blood ketone levels in non-obese control rats.

Group	Experimental Regimen	Mean ± Standard Error
Non-obese	IF-HFD	1.2± 0.1*^†^
	AL-HFD	1.1± 0.1^‡^
	IF-SD	0.8± 0.1
	AL-SD	0.7± 0.1

* Significant difference of *p*<0.05 compared to IF-SD

† Significant difference of *p*<0.01 compared to AL-SD

‡ Significant difference of *p*<0.05 compared to AL-SD; Intermittent fasting (IF); *ad libitum* (AL); high-fat diet (HFD); standard diet (SD).

**Table 4:** Blood ketone levels in obese rats.

Group	Experimental Regimen	Mean ± Standard Error
Obese	IF-HFD	0.5± 0.1
	AL-HFD	0.8± 0.1^‡^
	IF-SD	0.8± 0.1^†^
	AL-SD	0.3± 0.2

‡ = Significant difference of *p*<0.05 compared to AL-SD

† = Significant difference of *p*<0.01 compared to AL-SD; Intermittent fasting (IF); *ad libitum* (AL); high-fat diet (HFD); standard diet (SD).

### Behavioral data

**Fig 4:**
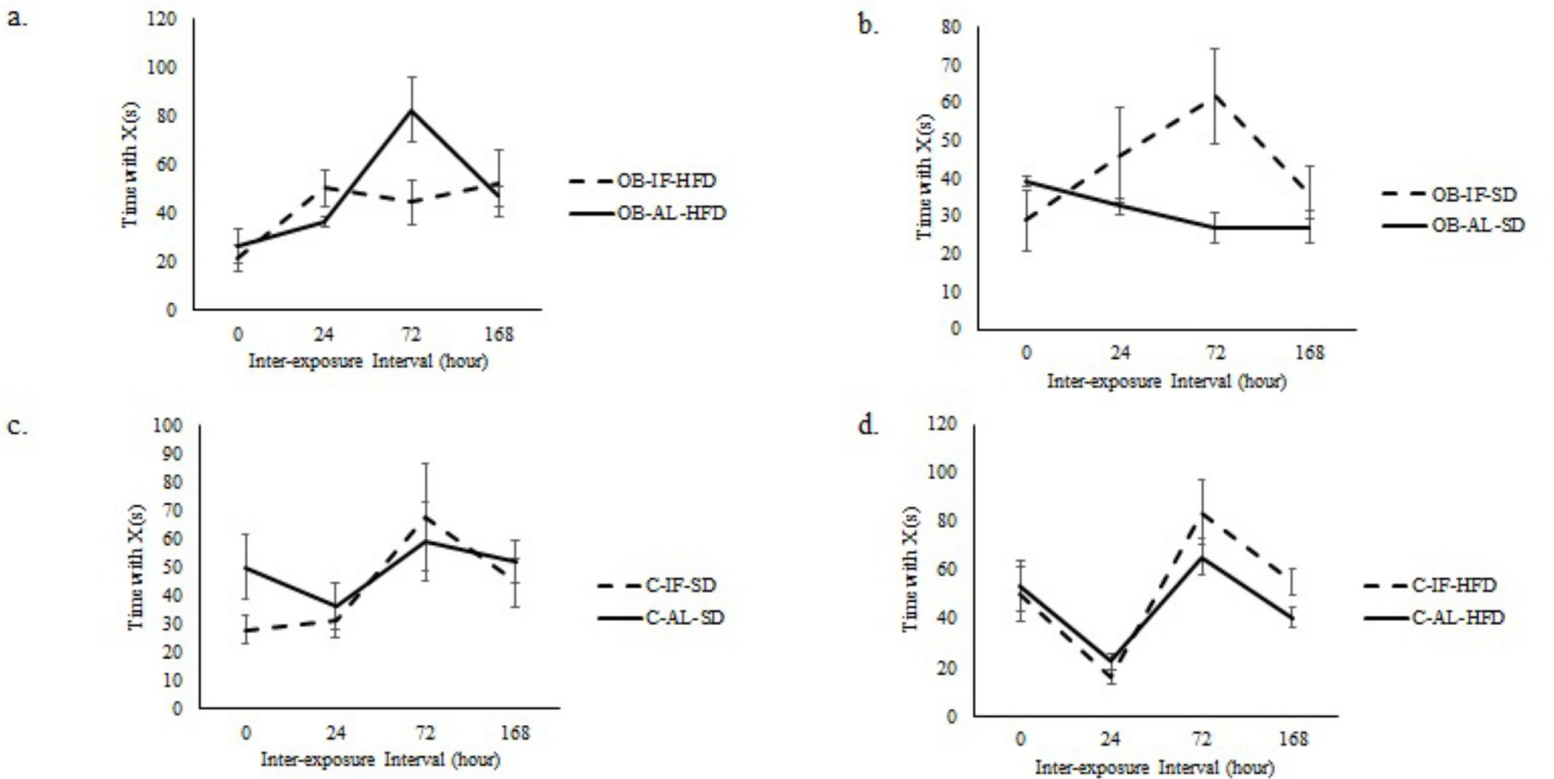
Time spent with novel objects. Obese (OB); Non-obese controls (C); IF: intermittent fasting; AL: *ad libitum*; HFD: high-fat diet; SD: standard diet. Error bars represent standard error.

Although there was no significant difference in the time spent with novel versus familiar objects in NOR testing or in the OF measurements between IF and AL groups in non-obese or obese rats (Tables 5 and 6; Fig 4), higher blood ketone levels correlated with greater distance traveled in both IF and AL groups (*p*<0.05; Table 7).

**Table 5:** Open field measurements in Non-obese control rats.

Non-obese Rats		
	AL	IF
	Mean ± Standard Error	Mean ± Standard Error
Distance	4.2± 0.2	4.0± 0.2
Line Crossings	240.6 ± 11.5	224.9 ± 8.0
Mean Speed	0.09 ± 0.01	0.08 ± 0.01
Middle Zone Distance Traveled	0.8 ± 0.1	0.7 ± 0.05
Time Spent in Middle Zone	41.5 ± 4.0	41.8 ± 3.3
Wall Zone Distance Traveled	2.9 ± 0.2	2.7 ± 0.1
Time Spent in Wall Zone	291.1 ± 4.9	292 ± 4.9

*Ad libitum* (AL); Intermittent fasting (IF).

**Table 6:** Open field measurements in obese rats.

Obese Rats		
	AL	IF
	Mean ± Standard Error	Mean ± Standard Error
Distance	3.6 ± 0.13	3.7 ± 0.15
Line Crossings	228.9 ± 19.5	222.6 ± 5.8
Mean Speed	0.06 ± 0.01	0.06 ± 0.01
Middle Zone Distance Traveled	0.7 ± 0.1	0.7 ± 0.06
Time Spent in Middle Zone	40.5 ± 6.2	41.8 ± 4.6
Wall Zone Distance Traveled	2.7 ± 0.2	2.6 ± 0.1
Time Spent in Wall Zone	291.2 ± 8	287.7 ± 5.8

*Ad libitum* (AL); Intermittent fasting (IF).

**Table 7:** Ketone correlation matrix including all rat groups.

		Distance Traveled (m)	Time with A	Time with X
**Ketone (mmol/L)**	Correlation	0.215	-0.089	-0.120
	p-value	0.026*	0.546	0.415

* = *p*<0.05. Time with A: time with familiar object; Time with X: time with novel object.

## Discussion

This study utilized a DIO model to examine whether IF would result in mental and physical fatigue in obese and non-obese rats. It was found that IF, regardless of diet, led to decreased weight gain and lower blood glucose levels [13, 44, 45]. These findings are supported by Bhoumik et al. (2020), which used Wistar rats to evaluate the effects of a time-restricted feeding model of IF (18- hour fast, 6- hour fed) or alternate day fasting (24 hours fed, 24 hours fasted) compared to AL controls over a 4-week time period. At the end of the study, it was found that both time-restricted feeding and alternate-day fasting resulted in decreased body weight and lower fasting blood glucose levels compared to AL rats [44]. In a study by Spezani et al. (2020), male C57BL/6 mice were exposed to IF (alternating between 24-hour access to food and 24-hour without access to food) for a period of 4 weeks while being fed either a standard (10% kcal fat), high-fat (50% kcal fat), or high-sucrose (50% kcal sucrose) diet. After 4 weeks on the IF regimen with these diets, all the mice exhibited weight loss and lower fasting glucose levels [13].

Weight loss and lower glucose levels are often associated with the metabolic switch that occurs during an IF regimen, which results in an increase in BHB levels due to lipid metabolism [10, 12]. In this study, obese rats fed SD on IF had higher ketone levels than their AL-SD counterparts. The group that exhibited the greatest difference in blood ketone levels were the C-AL-HFD and C-IF-HFD groups. In a study by Dedaul et al. (2019) using non-obese male C57BL/6 mice that were fed HFD fasted for 8 hours a day for 4 days or fed AL, IF alone increased beta-oxidation but HFD paired with IF further increased this process, thus resulting in higher ketone levels [46]. It was shown that mice fed HFD-IF had greater metabolic flexibility as evidenced by the increase in phosphorylation of lipid metabolism regulators and greater ability to activate lipolysis in white adipose tissue [46].

While differences in ketone levels were not significantly different between all of the IF groups compared to AL groups (Fig 1), higher ketone levels, whether induced by diet or the IF regimen, were correlated with increased distance traveled during OF, indicating a resistance to physical fatigue. Ketones provide an alternative fuel for oxidative phosphorylation and makes oxidation a preferential process, which minimizes glycolysis [47]. Increased ketone levels have been associated with improved physical performance and decreased fatigue in previous studies [23, 24]. A study by Nozawa et al. (2009) demonstrated the benefits of increased blood ketone levels in combating physical fatigue with mice exposed to bonito extract, an agent that increases ketone levels. Mice exposed to bonito extract were put through a forced swimming test and forced walking model to test for physical fatigue. Mice on bonito extract exhibited increased ketone levels as expected and resistance to physical fatigue [23]. This finding is further supported by a study by Murray et al. (2016) where rats fed a 30% ketone diet produced higher ketone levels and ran 32% further than control rats during a treadmill walking test [24].

Similar to OF testing results, the NOR testing results showed that IF had no negative impact on recognition memory which is an indirect measure of cognition. While many studies have reported improved cognition with IF regimens, these studies utilized longer duration of IF [30, 48, 49]. A study by Elesawy et al. (2021) saw improvements in cognition via elevated plus maze testing after 12 weeks of IF (16-hour daily fast). It was reported that IF rats had an increase in BDNF and neurotrophin-3, which they contributed as the factors improving cognition [49]. A study by Anson et al. (2003) utilized an alternate day fasting model in C57BL/6 male mice over a period of 20 weeks and measured IGF-1 signaling as evidence of neuroprotective qualities. It was found that IF rats had higher levels of IGF-1 signaling compared to AL groups, suggesting that IF may have a beneficial effect on cognition [48]. An improvement in cognition was also demonstrated in a study by Li et al. (2013). After exposing 7-week-old mice to control, HFD, or alternate-day fasting with SD conditions over 11 months, the animals underwent a Barnes maze test to measure spatial working memory by recording the amount of time it took to enter the correct target box that was previously introduced during a habituation phase. The mice in the fasting condition outperformed mice in the other groups [30].

One limitation of this study was the shortened duration of exposure to IF. Though 2 weeks of exposure produced noticeable differences in body weight and glucose levels, it is possible that extending the duration of IF could enhance the results of behavioral tests as well as ketone levels. The IF regimen in our study did not cause a significant increase in the ketone levels in the OB-IF-HFD versus OB-AL-HFD group or the C-IF-SD versus C-AL-SD groups, potentially due to the shorter duration of IF. Other studies have shown increases in ketone levels when exposing rodents to IF over a long-term duration [48, 50]. A study by Anson et al. (2003) utilized an alternate day fasting regimen in male C57BL/6 mice for a period of 20 weeks. By the end of the study, these mice exhibited decreased glucose levels and increased plasma ketone levels while the body weight was maintained throughout the study [48]. These findings were reiterated in a study by Park et al. (2020) in an 8-week study of rats fed either a ketogenic diet, 30% HFD, IF (24 hours fed, 24 hours fasted), high carbohydrate, or control diet. Rats on IF had higher plasma ketone levels and decreased body weight, but did not exhibit a difference in fasting glucose levels [50]. Additionally, measuring food consumption and calculating caloric intake could be used to further explain the observations from this study. Future studies should include food intake measurements and an extended timeline to overcome these limitations.

In conclusion, this study validates the use of IF for improved fasting glucose levels and decreased weight gain irrespective of the nature of the diet in both non-obese and obese groups. IF for 2 weeks does not contribute to mental or physical fatigue but longer duration may offer more benefits. Furthermore, increased ketone levels were correlated with increased physical activity, suggesting a protective role of ketones against physical fatigue.

## Supporting information

S1 File (RTF)

S1 Graphical abstract (TXT)
