# Supplementary material for: Intermittent fasting and mental and physical fatigue in obese and non-obese rats
Source: PLoS One. 2022 Nov 2;17(11):e0275684. doi: 10.1371/journal.pone.0275684 (PMC9629590; doi:10.1371/journal.pone.0275684)
Supplement: S1 File — (RTF) [file pone.0275684.s001.rtf]

Model Information	
Data Set	WORK.WEEKLY	
Dependent Variable	WkWt	
Covariance Structure	Unstructured	
Subject Effect	Animal	
Estimation Method	REML	
Residual Variance Method	None	
Fixed Effects SE Method	Model-Based	
Degrees of Freedom Method	Between-Within	


Class Level Information	
Class	Levels	Values	
Animal	26	C1 C10 C11 C12 C13 C14 C15 C16 C17 C18 C19 C2 C20 C21 C22 C23 C24 C25 C26 C3 C4 C5 C6 C7 C8 C9	
Group	2	IF ad_lib	
Diet2	2	HFD SD	
semester	3	fall spring summer	
Week	4	1 2 3 4	


Dimensions	
Covariance Parameters	10	
Columns in X	17	
Columns in Z	0	
Subjects	26	
Max Obs per Subject	4	


Number of Observations	
Number of Observations Read	101	
Number of Observations Used	87	
Number of Observations Not Used	14	


Iteration History	
Iteration	Evaluations	-2 Res Log Like	Criterion	
0	1	724.70164311		
1	2	626.34354904	0.00085444	
2	2	623.51269815	0.00060924	
3	2	621.66386511	0.00061161	
4	1	619.77498803	0.01396980	
5	1	616.76196243	0.00880704	
6	1	614.07154569	0.00533097	
7	1	612.59487852	0.00399368	
8	1	611.32844608	0.00355337	
9	1	610.59807508	0.00316053	
10	1	609.62771034	0.00233569	
11	1	608.99058558	0.00182000	
12	1	608.42079034	0.00145039	
13	1	608.04696157	0.00104480	
14	1	607.72799489	0.00068868	
15	1	607.54452171	0.00040911	
16	1	607.42259272	0.00019753	
17	1	607.36854597	0.00007163	
18	1	607.34846618	0.00001394	
19	1	607.34484543	0.00000083	
20	1	607.34464574	0.00000000	


Convergence criteria met.	


Covariance Parameter Estimates	
Cov Parm	Subject	Estimate	
UN(1,1)	Animal	362.48	
UN(2,1)	Animal	418.11	
UN(2,2)	Animal	740.28	
UN(3,1)	Animal	432.24	
UN(3,2)	Animal	728.69	
UN(3,3)	Animal	751.17	
UN(4,1)	Animal	479.68	
UN(4,2)	Animal	972.17	
UN(4,3)	Animal	991.22	
UN(4,4)	Animal	1396.79	


Fit Statistics	
-2 Res Log Likelihood	607.3	
AIC (Smaller is Better)	627.3	
AICC (Smaller is Better)	630.6	
BIC (Smaller is Better)	639.9	


Null Model Likelihood Ratio Test	
DF	Chi-Square	Pr > ChiSq	
9	117.36	<.0001	


Type 3 Tests of Fixed Effects	
Effect	Num DF	Den DF	F Value	Pr > F	
Group	1	23	8.95	0.0065	
Diet2	1	23	21.02	0.0001	
Week	3	23	100.05	<.0001	
Group*Week	3	23	3.36	0.0363	


Least Squares Means	
Effect	Group	Week	Estimate	Standard
Error	DF	t Value	Pr > |t|	
Group	IF		374.05	7.4748	23	50.04	<.0001	
Group	ad_lib		405.45	7.3798	23	54.94	<.0001	
Group*Week	IF	1	351.63	5.2878	23	66.50	<.0001	
Group*Week	IF	2	371.23	7.6617	23	48.45	<.0001	
Group*Week	IF	3	381.36	7.7074	23	49.48	<.0001	
Group*Week	IF	4	391.98	10.6813	23	36.70	<.0001	
Group*Week	ad_lib	1	375.01	5.2878	23	70.92	<.0001	
Group*Week	ad_lib	2	405.49	7.5513	23	53.70	<.0001	
Group*Week	ad_lib	3	414.40	7.6066	23	54.48	<.0001	
Group*Week	ad_lib	4	426.90	10.4293	23	40.93	<.0001	


Differences of Least Squares Means	
Effect	Group	Week	_Group	_Week	Estimate	Standard
Error	DF	t Value	Pr > |t|	
Group	IF		ad_lib		-31.4016	10.4949	23	-2.99	0.0065	
Group*Week	IF	1	IF	2	-19.5915	4.7073	23	-4.16	0.0004	
Group*Week	IF	1	IF	3	-29.7228	4.5477	23	-6.54	<.0001	
Group*Week	IF	1	IF	4	-40.3483	8.2481	23	-4.89	<.0001	
Group*Week	IF	1	ad_lib	1	-23.3748	7.4676	23	-3.13	0.0047	
Group*Week	IF	1	ad_lib	2	-53.8604	9.2102	23	-5.85	<.0001	
Group*Week	IF	1	ad_lib	3	-62.7666	9.2556	23	-6.78	<.0001	
Group*Week	IF	1	ad_lib	4	-75.2672	11.6859	23	-6.44	<.0001	
Group*Week	IF	2	IF	3	-10.1312	1.6838	23	-6.02	<.0001	
Group*Week	IF	2	IF	4	-20.7567	4.1952	23	-4.95	<.0001	
Group*Week	IF	2	ad_lib	1	-3.7832	9.2996	23	-0.41	0.6879	
Group*Week	IF	2	ad_lib	2	-34.2688	10.7492	23	-3.19	0.0041	
Group*Week	IF	2	ad_lib	3	-43.1751	10.7881	23	-4.00	0.0006	
Group*Week	IF	2	ad_lib	4	-55.6757	12.9335	23	-4.30	0.0003	
Group*Week	IF	3	IF	4	-10.6255	3.9188	23	-2.71	0.0125	
Group*Week	IF	3	ad_lib	1	6.3480	9.3370	23	0.68	0.5034	
Group*Week	IF	3	ad_lib	2	-24.1376	10.7815	23	-2.24	0.0351	
Group*Week	IF	3	ad_lib	3	-33.0438	10.8203	23	-3.05	0.0056	
Group*Week	IF	3	ad_lib	4	-45.5444	12.9603	23	-3.51	0.0019	
Group*Week	IF	4	ad_lib	1	16.9735	11.9091	23	1.43	0.1675	
Group*Week	IF	4	ad_lib	2	-13.5121	13.0725	23	-1.03	0.3121	
Group*Week	IF	4	ad_lib	3	-22.4184	13.1045	23	-1.71	0.1006	
Group*Week	IF	4	ad_lib	4	-34.9189	14.9203	23	-2.34	0.0283	
Group*Week	ad_lib	1	ad_lib	2	-30.4856	4.5280	23	-6.73	<.0001	
Group*Week	ad_lib	1	ad_lib	3	-39.3919	4.3780	23	-9.00	<.0001	
Group*Week	ad_lib	1	ad_lib	4	-51.8924	7.9223	23	-6.55	<.0001	
Group*Week	ad_lib	2	ad_lib	3	-8.9063	1.6188	23	-5.50	<.0001	
Group*Week	ad_lib	2	ad_lib	4	-21.4068	4.0071	23	-5.34	<.0001	
Group*Week	ad_lib	3	ad_lib	4	-12.5006	3.7367	23	-3.35	0.0028	

Model Information	
Data Set	WORK.WEEKLY	
Dependent Variable	WkWt	
Covariance Structure	Unstructured	
Subject Effect	Animal	
Estimation Method	REML	
Residual Variance Method	None	
Fixed Effects SE Method	Model-Based	
Degrees of Freedom Method	Between-Within	


Class Level Information	
Class	Levels	Values	
Animal	22	T1 T10 T11 T12 T13 T14 T15 T17 T2 T21 T22 T23 T24 T26 T27 T3 T4 T5 T6 T7 T8 T9	
Group	2	IF ad_lib	
Diet2	2	HFD SD	
semester	3	fall spring summer	
Week	4	1 2 3 4	


Dimensions	
Covariance Parameters	10	
Columns in X	17	
Columns in Z	0	
Subjects	22	
Max Obs per Subject	4	


Number of Observations	
Number of Observations Read	88	
Number of Observations Used	86	
Number of Observations Not Used	2	


Iteration History	
Iteration	Evaluations	-2 Res Log Like	Criterion	
0	1	722.77380759		
1	2	571.59075717	0.19103546	
2	1	564.68615130	0.06040272	
3	3	563.24275631	0.01725025	
4	3	560.93285130	0.00477478	
5	1	559.72411394	0.00089149	
6	1	559.51278312	0.00005163	
7	1	559.50154750	0.00000022	
8	1	559.50150141	0.00000000	


Convergence criteria met.	


Covariance Parameter Estimates	
Cov Parm	Subject	Estimate	
UN(1,1)	Animal	518.25	
UN(2,1)	Animal	441.38	
UN(2,2)	Animal	465.98	
UN(3,1)	Animal	469.89	
UN(3,2)	Animal	505.64	
UN(3,3)	Animal	573.22	
UN(4,1)	Animal	429.62	
UN(4,2)	Animal	500.54	
UN(4,3)	Animal	583.84	
UN(4,4)	Animal	628.41	


Fit Statistics	
-2 Res Log Likelihood	559.5	
AIC (Smaller is Better)	579.5	
AICC (Smaller is Better)	582.8	
BIC (Smaller is Better)	590.4	


Null Model Likelihood Ratio Test	
DF	Chi-Square	Pr > ChiSq	
9	163.27	<.0001	


Type 3 Tests of Fixed Effects	
Effect	Num DF	Den DF	F Value	Pr > F	
Group	1	19	3.54	0.0754	
Diet2	1	19	0.00	0.9614	
Week	3	19	52.19	<.0001	
Group*Week	3	19	5.70	0.0059	


Least Squares Means	
Effect	Group	Week	Estimate	Standard
Error	DF	t Value	Pr > |t|	
Group	IF		401.71	6.5101	19	61.71	<.0001	
Group	ad_lib		419.99	7.1393	19	58.83	<.0001	
Group*Week	IF	1	390.09	6.6071	19	59.04	<.0001	
Group*Week	IF	2	393.88	6.2689	19	62.83	<.0001	
Group*Week	IF	3	408.53	6.9452	19	58.82	<.0001	
Group*Week	IF	4	414.33	7.2740	19	56.96	<.0001	
Group*Week	ad_lib	1	397.77	7.2455	19	54.90	<.0001	
Group*Week	ad_lib	2	416.84	6.8754	19	60.63	<.0001	
Group*Week	ad_lib	3	428.91	7.6154	19	56.32	<.0001	
Group*Week	ad_lib	4	436.43	7.9768	19	54.71	<.0001	


Differences of Least Squares Means	
Effect	Group	Week	_Group	_Week	Estimate	Standard
Error	DF	t Value	Pr > |t|	
Group	IF		ad_lib		-18.2772	9.7187	19	-1.88	0.0754	
Group*Week	IF	1	IF	2	-3.7929	2.9078	19	-1.30	0.2077	
Group*Week	IF	1	IF	3	-18.4402	3.5553	19	-5.19	<.0001	
Group*Week	IF	1	IF	4	-24.2338	4.9050	19	-4.94	<.0001	
Group*Week	IF	1	ad_lib	1	-7.6737	9.8627	19	-0.78	0.4461	
Group*Week	IF	1	ad_lib	2	-26.7458	9.5940	19	-2.79	0.0117	
Group*Week	IF	1	ad_lib	3	-38.8187	10.1375	19	-3.83	0.0011	
Group*Week	IF	1	ad_lib	4	-46.3372	10.4100	19	-4.45	0.0003	
Group*Week	IF	2	IF	3	-14.6474	1.5254	19	-9.60	<.0001	
Group*Week	IF	2	IF	4	-20.4409	2.8079	19	-7.28	<.0001	
Group*Week	IF	2	ad_lib	1	-3.8808	9.6393	19	-0.40	0.6917	
Group*Week	IF	2	ad_lib	2	-22.9530	9.3643	19	-2.45	0.0241	
Group*Week	IF	2	ad_lib	3	-35.0259	9.9204	19	-3.53	0.0022	
Group*Week	IF	2	ad_lib	4	-42.5444	10.1986	19	-4.17	0.0005	
Group*Week	IF	3	IF	4	-5.7936	1.7141	19	-3.38	0.0031	
Group*Week	IF	3	ad_lib	1	10.7665	10.0922	19	1.07	0.2994	
Group*Week	IF	3	ad_lib	2	-8.3056	9.8299	19	-0.84	0.4087	
Group*Week	IF	3	ad_lib	3	-20.3785	10.3610	19	-1.97	0.0640	
Group*Week	IF	3	ad_lib	4	-27.8970	10.6277	19	-2.62	0.0167	
Group*Week	IF	4	ad_lib	1	16.5601	10.3195	19	1.60	0.1250	
Group*Week	IF	4	ad_lib	2	-2.5120	10.0631	19	-0.25	0.8056	
Group*Week	IF	4	ad_lib	3	-14.5850	10.5825	19	-1.38	0.1842	
Group*Week	IF	4	ad_lib	4	-22.1034	10.8438	19	-2.04	0.0557	
Group*Week	ad_lib	1	ad_lib	2	-19.0721	3.1854	19	-5.99	<.0001	
Group*Week	ad_lib	1	ad_lib	3	-31.1450	3.8947	19	-8.00	<.0001	
Group*Week	ad_lib	1	ad_lib	4	-38.6635	5.3759	19	-7.19	<.0001	
Group*Week	ad_lib	2	ad_lib	3	-12.0729	1.6710	19	-7.22	<.0001	
Group*Week	ad_lib	2	ad_lib	4	-19.5914	3.0806	19	-6.36	<.0001	
Group*Week	ad_lib	3	ad_lib	4	-7.5185	1.8854	19	-3.99	0.0008	

Model Information	
Data Set	WORK.WEEKLY	
Dependent Variable	WkGlucose	
Covariance Structure	Unstructured	
Subject Effect	Animal	
Estimation Method	REML	
Residual Variance Method	None	
Fixed Effects SE Method	Model-Based	
Degrees of Freedom Method	Between-Within	


Class Level Information	
Class	Levels	Values	
Animal	26	C1 C10 C11 C12 C13 C14 C15 C16 C17 C18 C19 C2 C20 C21 C22 C23 C24 C25 C26 C3 C4 C5 C6 C7 C8 C9	
Group	2	IF ad_lib	
Diet2	2	HFD SD	
semester	3	fall spring summer	
Week	4	1 2 3 4	


Dimensions	
Covariance Parameters	10	
Columns in X	17	
Columns in Z	0	
Subjects	26	
Max Obs per Subject	4	


Number of Observations	
Number of Observations Read	101	
Number of Observations Used	101	
Number of Observations Not Used	0	


Iteration History	
Iteration	Evaluations	-2 Res Log Like	Criterion	
0	1	653.42653425		
1	2	634.04578839	0.00000047	
2	1	634.04567973	0.00000000	


Convergence criteria met.	


Covariance Parameter Estimates	
Cov Parm	Subject	Estimate	
UN(1,1)	Animal	41.7990	
UN(2,1)	Animal	2.4524	
UN(2,2)	Animal	28.8834	
UN(3,1)	Animal	6.1816	
UN(3,2)	Animal	18.2225	
UN(3,3)	Animal	59.7506	
UN(4,1)	Animal	25.4012	
UN(4,2)	Animal	0.6484	
UN(4,3)	Animal	18.3631	
UN(4,4)	Animal	90.9605	


Fit Statistics	
-2 Res Log Likelihood	634.0	
AIC (Smaller is Better)	654.0	
AICC (Smaller is Better)	656.8	
BIC (Smaller is Better)	666.6	


Null Model Likelihood Ratio Test	
DF	Chi-Square	Pr > ChiSq	
9	19.38	0.0221	


Type 3 Tests of Fixed Effects	
Effect	Num DF	Den DF	F Value	Pr > F	
Group	1	23	30.39	<.0001	
Diet2	1	23	7.02	0.0143	
Week	3	23	0.65	0.5882	
Group*Week	3	23	2.40	0.0942	


Least Squares Means	
Effect	Group	Week	Estimate	Standard
Error	DF	t Value	Pr > |t|	
Group	IF		88.9916	1.3569	23	65.58	<.0001	
Group	ad_lib		99.4377	1.3243	23	75.09	<.0001	
Group*Week	IF	1	92.5428	1.7943	23	51.58	<.0001	
Group*Week	IF	2	89.3196	1.5511	23	57.58	<.0001	
Group*Week	IF	3	88.3202	2.2301	23	39.60	<.0001	
Group*Week	IF	4	85.7838	2.7354	23	31.36	<.0001	
Group*Week	ad_lib	1	98.7069	1.7943	23	55.01	<.0001	
Group*Week	ad_lib	2	98.7069	1.4919	23	66.16	<.0001	
Group*Week	ad_lib	3	98.8607	2.1448	23	46.09	<.0001	
Group*Week	ad_lib	4	101.48	2.6459	23	38.35	<.0001	


Differences of Least Squares Means	
Effect	Group	Week	_Group	_Week	Estimate	Standard
Error	DF	t Value	Pr > |t|	
Group	IF		ad_lib		-10.4461	1.8951	23	-5.51	<.0001	
Group*Week	IF	1	IF	2	3.2231	2.2908	23	1.41	0.1728	
Group*Week	IF	1	IF	3	4.2226	2.6908	23	1.57	0.1302	
Group*Week	IF	1	IF	4	6.7590	2.6056	23	2.59	0.0162	
Group*Week	IF	1	ad_lib	1	-6.1641	2.5359	23	-2.43	0.0233	
Group*Week	IF	1	ad_lib	2	-6.1641	2.3318	23	-2.64	0.0145	
Group*Week	IF	1	ad_lib	3	-6.3179	2.7949	23	-2.26	0.0336	
Group*Week	IF	1	ad_lib	4	-8.9333	3.1957	23	-2.80	0.0103	
Group*Week	IF	2	IF	3	0.9995	2.0849	23	0.48	0.6362	
Group*Week	IF	2	IF	4	3.5358	3.1304	23	1.13	0.2703	
Group*Week	IF	2	ad_lib	1	-9.3872	2.3717	23	-3.96	0.0006	
Group*Week	IF	2	ad_lib	2	-9.3872	2.1521	23	-4.36	0.0002	
Group*Week	IF	2	ad_lib	3	-9.5411	2.6468	23	-3.60	0.0015	
Group*Week	IF	2	ad_lib	4	-12.1565	3.0670	23	-3.96	0.0006	
Group*Week	IF	3	IF	4	2.5364	3.0729	23	0.83	0.4176	
Group*Week	IF	3	ad_lib	1	-10.3867	2.8621	23	-3.63	0.0014	
Group*Week	IF	3	ad_lib	2	-10.3867	2.6829	23	-3.87	0.0008	
Group*Week	IF	3	ad_lib	3	-10.5406	3.0939	23	-3.41	0.0024	
Group*Week	IF	3	ad_lib	4	-13.1559	3.4602	23	-3.80	0.0009	
Group*Week	IF	4	ad_lib	1	-12.9231	3.2706	23	-3.95	0.0006	
Group*Week	IF	4	ad_lib	2	-12.9231	3.1150	23	-4.15	0.0004	
Group*Week	IF	4	ad_lib	3	-13.0769	3.4753	23	-3.76	0.0010	
Group*Week	IF	4	ad_lib	4	-15.6923	3.8051	23	-4.12	0.0004	
Group*Week	ad_lib	1	ad_lib	2	-769E-12	2.2494	23	-0.00	1.0000	
Group*Week	ad_lib	1	ad_lib	3	-0.1538	2.6193	23	-0.06	0.9537	
Group*Week	ad_lib	1	ad_lib	4	-2.7692	2.5109	23	-1.10	0.2815	
Group*Week	ad_lib	2	ad_lib	3	-0.1538	2.0036	23	-0.08	0.9395	
Group*Week	ad_lib	2	ad_lib	4	-2.7692	3.0198	23	-0.92	0.3686	
Group*Week	ad_lib	3	ad_lib	4	-2.6154	2.9611	23	-0.88	0.3862	

Model Information	
Data Set	WORK.WEEKLY	
Dependent Variable	WkGlucose	
Covariance Structure	Unstructured	
Subject Effect	Animal	
Estimation Method	REML	
Residual Variance Method	None	
Fixed Effects SE Method	Model-Based	
Degrees of Freedom Method	Between-Within	


Class Level Information	
Class	Levels	Values	
Animal	22	T1 T10 T11 T12 T13 T14 T15 T17 T2 T21 T22 T23 T24 T26 T27 T3 T4 T5 T6 T7 T8 T9	
Group	2	IF ad_lib	
Diet2	2	HFD SD	
semester	3	fall spring summer	
Week	4	1 2 3 4	


Dimensions	
Covariance Parameters	10	
Columns in X	17	
Columns in Z	0	
Subjects	22	
Max Obs per Subject	4	


Number of Observations	
Number of Observations Read	88	
Number of Observations Used	88	
Number of Observations Not Used	0	


Iteration History	
Iteration	Evaluations	-2 Res Log Like	Criterion	
0	1	504.54393491		
1	2	498.04098663	0.00000079	
2	1	498.04084633	0.00000000	


Convergence criteria met.	


Covariance Parameter Estimates	
Cov Parm	Subject	Estimate	
UN(1,1)	Animal	30.6889	
UN(2,1)	Animal	5.3262	
UN(2,2)	Animal	20.6901	
UN(3,1)	Animal	-4.9319	
UN(3,2)	Animal	2.8176	
UN(3,3)	Animal	20.6708	
UN(4,1)	Animal	10.8975	
UN(4,2)	Animal	1.3243	
UN(4,3)	Animal	-2.3503	
UN(4,4)	Animal	33.5199	


Fit Statistics	
-2 Res Log Likelihood	498.0	
AIC (Smaller is Better)	518.0	
AICC (Smaller is Better)	521.3	
BIC (Smaller is Better)	529.0	


Null Model Likelihood Ratio Test	
DF	Chi-Square	Pr > ChiSq	
9	6.50	0.6887	


Type 3 Tests of Fixed Effects	
Effect	Num DF	Den DF	F Value	Pr > F	
Group	1	19	32.33	<.0001	
Diet2	1	19	13.45	0.0016	
Week	3	19	18.38	<.0001	
Group*Week	3	19	4.28	0.0182	


Least Squares Means	
Effect	Group	Week	Estimate	Standard
Error	DF	t Value	Pr > |t|	
Group	IF		89.6021	0.8341	19	107.42	<.0001	
Group	ad_lib		96.6942	0.9150	19	105.68	<.0001	
Group*Week	IF	1	96.3312	1.6022	19	60.12	<.0001	
Group*Week	IF	2	85.9840	1.3167	19	65.30	<.0001	
Group*Week	IF	3	89.0673	1.3161	19	67.67	<.0001	
Group*Week	IF	4	87.0257	1.6742	19	51.98	<.0001	
Group*Week	ad_lib	1	101.17	1.7558	19	57.62	<.0001	
Group*Week	ad_lib	2	98.7692	1.4432	19	68.44	<.0001	
Group*Week	ad_lib	3	96.0692	1.4425	19	66.60	<.0001	
Group*Week	ad_lib	4	90.7692	1.8346	19	49.48	<.0001	


Differences of Least Squares Means	
Effect	Group	Week	_Group	_Week	Estimate	Standard
Error	DF	t Value	Pr > |t|	
Group	IF		ad_lib		-7.0921	1.2473	19	-5.69	<.0001	
Group*Week	IF	1	IF	2	10.3472	1.8422	19	5.62	<.0001	
Group*Week	IF	1	IF	3	7.2639	2.2587	19	3.22	0.0045	
Group*Week	IF	1	IF	4	9.3056	1.8800	19	4.95	<.0001	
Group*Week	IF	1	ad_lib	1	-4.8380	2.3817	19	-2.03	0.0565	
Group*Week	IF	1	ad_lib	2	-2.4380	2.1617	19	-1.13	0.2734	
Group*Week	IF	1	ad_lib	3	0.2620	2.1612	19	0.12	0.9048	
Group*Week	IF	1	ad_lib	4	5.5620	2.4404	19	2.28	0.0344	
Group*Week	IF	2	IF	3	-3.0833	1.7254	19	-1.79	0.0899	
Group*Week	IF	2	IF	4	-1.0417	2.0729	19	-0.50	0.6211	
Group*Week	IF	2	ad_lib	1	-15.1852	2.1999	19	-6.90	<.0001	
Group*Week	IF	2	ad_lib	2	-12.7852	1.9595	19	-6.52	<.0001	
Group*Week	IF	2	ad_lib	3	-10.0852	1.9590	19	-5.15	<.0001	
Group*Week	IF	2	ad_lib	4	-4.7852	2.2633	19	-2.11	0.0479	
Group*Week	IF	3	IF	4	2.0417	2.2153	19	0.92	0.3683	
Group*Week	IF	3	ad_lib	1	-12.1019	2.1995	19	-5.50	<.0001	
Group*Week	IF	3	ad_lib	2	-9.7019	1.9591	19	-4.95	<.0001	
Group*Week	IF	3	ad_lib	3	-7.0019	1.9586	19	-3.57	0.0020	
Group*Week	IF	3	ad_lib	4	-1.7019	2.2630	19	-0.75	0.4612	
Group*Week	IF	4	ad_lib	1	-14.1435	2.4308	19	-5.82	<.0001	
Group*Week	IF	4	ad_lib	2	-11.7435	2.2156	19	-5.30	<.0001	
Group*Week	IF	4	ad_lib	3	-9.0435	2.2151	19	-4.08	0.0006	
Group*Week	IF	4	ad_lib	4	-3.7435	2.4883	19	-1.50	0.1489	
Group*Week	ad_lib	1	ad_lib	2	2.4000	2.0181	19	1.19	0.2490	
Group*Week	ad_lib	1	ad_lib	3	5.1000	2.4743	19	2.06	0.0532	
Group*Week	ad_lib	1	ad_lib	4	10.4000	2.0595	19	5.05	<.0001	
Group*Week	ad_lib	2	ad_lib	3	2.7000	1.8901	19	1.43	0.1694	
Group*Week	ad_lib	2	ad_lib	4	8.0000	2.2707	19	3.52	0.0023	
Group*Week	ad_lib	3	ad_lib	4	5.3000	2.4268	19	2.18	0.0417	

Model Information	
Data Set	WORK.WEEKLY	
Dependent Variable	WkKetone	
Covariance Structure	Unstructured	
Subject Effect	Animal	
Estimation Method	REML	
Residual Variance Method	None	
Fixed Effects SE Method	Model-Based	
Degrees of Freedom Method	Between-Within	


Class Level Information	
Class	Levels	Values	
Animal	26	C1 C10 C11 C12 C13 C14 C15 C16 C17 C18 C19 C2 C20 C21 C22 C23 C24 C25 C26 C3 C4 C5 C6 C7 C8 C9	
Group	2	IF ad_lib	
Diet2	2	HFD SD	
semester	3	fall spring summer	
Week	4	1 2 3 4	


Dimensions	
Covariance Parameters	10	
Columns in X	9	
Columns in Z	0	
Subjects	26	
Max Obs per Subject	4	


Number of Observations	
Number of Observations Read	101	
Number of Observations Used	101	
Number of Observations Not Used	0	


Iteration History	
Iteration	Evaluations	-2 Res Log Like	Criterion	
0	1	104.55201707		
1	2	35.31680542	0.00001368	
2	1	35.31581892	0.00000000	


Convergence criteria met.	


Covariance Parameter Estimates	
Cov Parm	Subject	Estimate	
UN(1,1)	Animal	0.1810	
UN(2,1)	Animal	0.1028	
UN(2,2)	Animal	0.1167	
UN(3,1)	Animal	0.1453	
UN(3,2)	Animal	0.1006	
UN(3,3)	Animal	0.1553	
UN(4,1)	Animal	0.1064	
UN(4,2)	Animal	0.09486	
UN(4,3)	Animal	0.1112	
UN(4,4)	Animal	0.1985	


Fit Statistics	
-2 Res Log Likelihood	35.3	
AIC (Smaller is Better)	55.3	
AICC (Smaller is Better)	57.9	
BIC (Smaller is Better)	67.9	


Null Model Likelihood Ratio Test	
DF	Chi-Square	Pr > ChiSq	
9	69.24	<.0001	


Type 3 Tests of Fixed Effects	
Effect	Num DF	Den DF	F Value	Pr > F	
Group	1	22	1.51	0.2323	
Diet2	1	22	10.27	0.0041	
Group*Diet2	1	22	0.01	0.9374	


Least Squares Means	
Effect	Group	Diet2	Estimate	Standard
Error	DF	t Value	Pr > |t|	
Diet2		HFD	1.1619	0.09624	22	12.07	<.0001	
Diet2		SD	0.7389	0.09039	22	8.17	<.0001	
Group*Diet2	IF	HFD	1.2377	0.1361	22	9.09	<.0001	
Group*Diet2	IF	SD	0.8252	0.1296	22	6.37	<.0001	
Group*Diet2	ad_lib	HFD	1.0861	0.1361	22	7.98	<.0001	
Group*Diet2	ad_lib	SD	0.6525	0.1260	22	5.18	<.0001	


Differences of Least Squares Means	
Effect	Group	Diet2	_Group	_Diet2	Estimate	Standard
Error	DF	t Value	Pr > |t|	
Diet2		HFD		SD	0.4230	0.1320	22	3.20	0.0041	
Group*Diet2	IF	HFD	IF	SD	0.4126	0.1880	22	2.19	0.0390	
Group*Diet2	IF	HFD	ad_lib	HFD	0.1517	0.1925	22	0.79	0.4391	
Group*Diet2	IF	HFD	ad_lib	SD	0.5852	0.1855	22	3.16	0.0046	
Group*Diet2	IF	SD	ad_lib	HFD	-0.2609	0.1880	22	-1.39	0.1791	
Group*Diet2	IF	SD	ad_lib	SD	0.1727	0.1808	22	0.96	0.3499	
Group*Diet2	ad_lib	HFD	ad_lib	SD	0.4335	0.1855	22	2.34	0.0289	

Model Information	
Data Set	WORK.WEEKLY	
Dependent Variable	WkKetone	
Covariance Structure	Unstructured	
Subject Effect	Animal	
Estimation Method	REML	
Residual Variance Method	None	
Fixed Effects SE Method	Model-Based	
Degrees of Freedom Method	Between-Within	


Class Level Information	
Class	Levels	Values	
Animal	22	T1 T10 T11 T12 T13 T14 T15 T17 T2 T21 T22 T23 T24 T26 T27 T3 T4 T5 T6 T7 T8 T9	
Group	2	IF ad_lib	
Diet2	2	HFD SD	
semester	3	fall spring summer	
Week	4	1 2 3 4	


Dimensions	
Covariance Parameters	10	
Columns in X	9	
Columns in Z	0	
Subjects	22	
Max Obs per Subject	4	


Number of Observations	
Number of Observations Read	88	
Number of Observations Used	88	
Number of Observations Not Used	0	


Iteration History	
Iteration	Evaluations	-2 Res Log Like	Criterion	
0	1	119.25553004		
1	4	51.66129764	0.00158665	
2	1	51.57162668	0.00004819	
3	1	51.56909478	0.00000006	
4	1	51.56909188	0.00000000	


Convergence criteria met.	


Covariance Parameter Estimates	
Cov Parm	Subject	Estimate	
UN(1,1)	Animal	0.1671	
UN(2,1)	Animal	0.1604	
UN(2,2)	Animal	0.2177	
UN(3,1)	Animal	0.1055	
UN(3,2)	Animal	0.1342	
UN(3,3)	Animal	0.1336	
UN(4,1)	Animal	0.2036	
UN(4,2)	Animal	0.1619	
UN(4,3)	Animal	0.1471	
UN(4,4)	Animal	0.4801	


Fit Statistics	
-2 Res Log Likelihood	51.6	
AIC (Smaller is Better)	71.6	
AICC (Smaller is Better)	74.6	
BIC (Smaller is Better)	82.5	


Null Model Likelihood Ratio Test	
DF	Chi-Square	Pr > ChiSq	
9	67.69	<.0001	


Type 3 Tests of Fixed Effects	
Effect	Num DF	Den DF	F Value	Pr > F	
Group	1	18	1.19	0.2896	
Diet2	1	18	0.26	0.6162	
Group*Diet2	1	18	8.11	0.0107	


Least Squares Means	
Effect	Group	Diet2	Estimate	Standard
Error	DF	t Value	Pr > |t|	
Diet2		HFD	0.6392	0.09826	18	6.51	<.0001	
Diet2		SD	0.5671	0.1017	18	5.58	<.0001	
Group*Diet2	IF	HFD	0.5150	0.1451	18	3.55	0.0023	
Group*Diet2	IF	SD	0.8457	0.1227	18	6.89	<.0001	
Group*Diet2	ad_lib	HFD	0.7635	0.1325	18	5.76	<.0001	
Group*Diet2	ad_lib	SD	0.2885	0.1623	18	1.78	0.0923	


Differences of Least Squares Means	
Effect	Group	Diet2	_Group	_Diet2	Estimate	Standard
Error	DF	t Value	Pr > |t|	
Diet2		HFD		SD	0.07214	0.1414	18	0.51	0.6162	
Group*Diet2	IF	HFD	IF	SD	-0.3307	0.1900	18	-1.74	0.0989	
Group*Diet2	IF	HFD	ad_lib	HFD	-0.2485	0.1965	18	-1.26	0.2221	
Group*Diet2	IF	HFD	ad_lib	SD	0.2264	0.2177	18	1.04	0.3121	
Group*Diet2	IF	SD	ad_lib	HFD	0.08216	0.1806	18	0.46	0.6545	
Group*Diet2	IF	SD	ad_lib	SD	0.5571	0.2034	18	2.74	0.0135	
Group*Diet2	ad_lib	HFD	ad_lib	SD	0.4750	0.2095	18	2.27	0.0359	

Model Information	
Data Set	WORK.TIME	
Dependent Variable	trans	
Covariance Structure	Unstructured	
Subject Effect	Animal	
Estimation Method	REML	
Residual Variance Method	None	
Fixed Effects SE Method	Model-Based	
Degrees of Freedom Method	Between-Within	


Class Level Information	
Class	Levels	Values	
Animal	25	C1 C10 C11 C12 C13 C14 C15 C16 C17 C18 C19 C20 C21 C22 C23 C24 C25 C26 C3 C4 C5 C6 C7 C8 C9	
Group	2	IF ad_lib	
Diet2	2	HFD SD	
TimeDay	4	0 1 3 7	


Dimensions	
Covariance Parameters	10	
Columns in X	21	
Columns in Z	0	
Subjects	25	
Max Obs per Subject	4	


Number of Observations	
Number of Observations Read	100	
Number of Observations Used	99	
Number of Observations Not Used	1	


Iteration History	
Iteration	Evaluations	-2 Res Log Like	Criterion	
0	1	170.43138233		
1	2	146.64347479	0.00000334	
2	1	146.64344647	0.00000000	


Convergence criteria met.	


Covariance Parameter Estimates	
Cov Parm	Subject	Estimate	
UN(1,1)	Animal	0.4967	
UN(2,1)	Animal	-0.03101	
UN(2,2)	Animal	0.1651	
UN(3,1)	Animal	-0.1895	
UN(3,2)	Animal	0.05744	
UN(3,3)	Animal	0.3453	
UN(4,1)	Animal	-0.05308	
UN(4,2)	Animal	0.03880	
UN(4,3)	Animal	0.1384	
UN(4,4)	Animal	0.1946	


Fit Statistics	
-2 Res Log Likelihood	146.6	
AIC (Smaller is Better)	166.6	
AICC (Smaller is Better)	169.5	
BIC (Smaller is Better)	178.8	


Null Model Likelihood Ratio Test	
DF	Chi-Square	Pr > ChiSq	
9	23.79	0.0046	


Type 3 Tests of Fixed Effects	
Effect	Num DF	Den DF	F Value	Pr > F	
Diet2	1	21	13.05	0.0016	
Group	1	21	4.61	0.0437	
TimeDay	3	21	43.77	<.0001	
Diet2*TimeDay	3	21	18.06	<.0001	
Group*Diet2	1	21	0.18	0.6718	


Least Squares Means	
Effect	Group	Diet2	TimeDay	Estimate	Standard
Error	DF	t Value	Pr > |t|	
Diet2*TimeDay		HFD	0	1.4509	0.2108	21	6.88	<.0001	
Diet2*TimeDay		HFD	1	-0.6566	0.1173	21	-5.60	<.0001	
Diet2*TimeDay		HFD	3	0.9799	0.1696	21	5.78	<.0001	
Diet2*TimeDay		HFD	7	0.6561	0.1273	21	5.15	<.0001	
Diet2*TimeDay		SD	0	0.1950	0.1956	21	1.00	0.3299	
Diet2*TimeDay		SD	1	-0.05841	0.1128	21	-0.52	0.6102	
Diet2*TimeDay		SD	3	0.4440	0.1631	21	2.72	0.0128	
Diet2*TimeDay		SD	7	0.3016	0.1225	21	2.46	0.0225	
Group	IF			0.3011	0.07705	21	3.91	0.0008	
Group	ad_lib			0.5270	0.07308	21	7.21	<.0001	


Differences of Least Squares Means	
Effect	Group	Diet2	TimeDay	_Group	_Diet2	_TimeDay	Estimate	Standard
Error	DF	t Value	Pr > |t|	
Diet2*TimeDay		HFD	0		HFD	1	2.1076	0.2517	21	8.37	<.0001	
Diet2*TimeDay		HFD	0		HFD	3	0.4711	0.3237	21	1.46	0.1604	
Diet2*TimeDay		HFD	0		HFD	7	0.7948	0.2636	21	3.01	0.0066	
Diet2*TimeDay		HFD	0		SD	0	1.2559	0.2875	21	4.37	0.0003	
Diet2*TimeDay		HFD	0		SD	1	1.5093	0.2391	21	6.31	<.0001	
Diet2*TimeDay		HFD	0		SD	3	1.0069	0.2665	21	3.78	0.0011	
Diet2*TimeDay		HFD	0		SD	7	1.1493	0.2438	21	4.71	0.0001	
Diet2*TimeDay		HFD	1		HFD	3	-1.6365	0.1816	21	-9.01	<.0001	
Diet2*TimeDay		HFD	1		HFD	7	-1.3128	0.1533	21	-8.56	<.0001	
Diet2*TimeDay		HFD	1		SD	0	-0.8517	0.2280	21	-3.73	0.0012	
Diet2*TimeDay		HFD	1		SD	1	-0.5982	0.1628	21	-3.68	0.0014	
Diet2*TimeDay		HFD	1		SD	3	-1.1007	0.2009	21	-5.48	<.0001	
Diet2*TimeDay		HFD	1		SD	7	-0.9583	0.1696	21	-5.65	<.0001	
Diet2*TimeDay		HFD	3		HFD	7	0.3237	0.1481	21	2.19	0.0402	
Diet2*TimeDay		HFD	3		SD	0	0.7848	0.2589	21	3.03	0.0063	
Diet2*TimeDay		HFD	3		SD	1	1.0383	0.2037	21	5.10	<.0001	
Diet2*TimeDay		HFD	3		SD	3	0.5358	0.2353	21	2.28	0.0334	
Diet2*TimeDay		HFD	3		SD	7	0.6782	0.2092	21	3.24	0.0039	
Diet2*TimeDay		HFD	7		SD	0	0.4611	0.2334	21	1.98	0.0614	
Diet2*TimeDay		HFD	7		SD	1	0.7145	0.1701	21	4.20	0.0004	
Diet2*TimeDay		HFD	7		SD	3	0.2121	0.2069	21	1.03	0.3169	
Diet2*TimeDay		HFD	7		SD	7	0.3545	0.1767	21	2.01	0.0578	
Diet2*TimeDay		SD	0		SD	1	0.2535	0.2360	21	1.07	0.2950	
Diet2*TimeDay		SD	0		SD	3	-0.2490	0.3065	21	-0.81	0.4257	
Diet2*TimeDay		SD	0		SD	7	-0.1066	0.2477	21	-0.43	0.6713	
Diet2*TimeDay		SD	1		SD	3	-0.5024	0.1744	21	-2.88	0.0090	
Diet2*TimeDay		SD	1		SD	7	-0.3600	0.1473	21	-2.44	0.0234	
Diet2*TimeDay		SD	3		SD	7	0.1424	0.1422	21	1.00	0.3282	
Group	IF			ad_lib			-0.2259	0.1052	21	-2.15	0.0437	

Differences of Least Squares Means	
Effect	Group	Diet2	TimeDay	_Group	_Diet2	_TimeDay	Adjustment	Adj P	
Diet2*TimeDay		HFD	0		HFD	1	Tukey-Kramer	<.0001	
Diet2*TimeDay		HFD	0		HFD	3	Tukey-Kramer	0.8215	
Diet2*TimeDay		HFD	0		HFD	7	Tukey-Kramer	0.0988	
Diet2*TimeDay		HFD	0		SD	0	Tukey-Kramer	0.0054	
Diet2*TimeDay		HFD	0		SD	1	Tukey-Kramer	<.0001	
Diet2*TimeDay		HFD	0		SD	3	Tukey-Kramer	0.0203	
Diet2*TimeDay		HFD	0		SD	7	Tukey-Kramer	0.0025	
Diet2*TimeDay		HFD	1		HFD	3	Tukey-Kramer	<.0001	
Diet2*TimeDay		HFD	1		HFD	7	Tukey-Kramer	<.0001	
Diet2*TimeDay		HFD	1		SD	0	Tukey-Kramer	0.0223	
Diet2*TimeDay		HFD	1		SD	1	Tukey-Kramer	0.0253	
Diet2*TimeDay		HFD	1		SD	3	Tukey-Kramer	0.0004	
Diet2*TimeDay		HFD	1		SD	7	Tukey-Kramer	0.0003	
Diet2*TimeDay		HFD	3		HFD	7	Tukey-Kramer	0.3976	
Diet2*TimeDay		HFD	3		SD	0	Tukey-Kramer	0.0956	
Diet2*TimeDay		HFD	3		SD	1	Tukey-Kramer	0.0010	
Diet2*TimeDay		HFD	3		SD	3	Tukey-Kramer	0.3500	
Diet2*TimeDay		HFD	3		SD	7	Tukey-Kramer	0.0630	
Diet2*TimeDay		HFD	7		SD	0	Tukey-Kramer	0.5191	
Diet2*TimeDay		HFD	7		SD	1	Tukey-Kramer	0.0079	
Diet2*TimeDay		HFD	7		SD	3	Tukey-Kramer	0.9650	
Diet2*TimeDay		HFD	7		SD	7	Tukey-Kramer	0.5007	
Diet2*TimeDay		SD	0		SD	1	Tukey-Kramer	0.9555	
Diet2*TimeDay		SD	0		SD	3	Tukey-Kramer	0.9904	
Diet2*TimeDay		SD	0		SD	7	Tukey-Kramer	0.9998	
Diet2*TimeDay		SD	1		SD	3	Tukey-Kramer	0.1276	
Diet2*TimeDay		SD	1		SD	7	Tukey-Kramer	0.2716	
Diet2*TimeDay		SD	3		SD	7	Tukey-Kramer	0.9692	
Group	IF			ad_lib				.	

Model Information	
Data Set	WORK.TIME	
Dependent Variable	trans	
Covariance Structure	Unstructured	
Subject Effect	Animal	
Estimation Method	REML	
Residual Variance Method	None	
Fixed Effects SE Method	Model-Based	
Degrees of Freedom Method	Between-Within	


Class Level Information	
Class	Levels	Values	
Animal	22	T1 T10 T11 T12 T13 T14 T15 T17 T2 T21 T22 T23 T24 T26 T27 T3 T4 T5 T6 T7 T8 T9	
Group	2	IF ad_lib	
Diet2	2	HFD SD	
TimeDay	4	0 1 3 7	


Dimensions	
Covariance Parameters	10	
Columns in X	21	
Columns in Z	0	
Subjects	22	
Max Obs per Subject	4	


Number of Observations	
Number of Observations Read	88	
Number of Observations Used	88	
Number of Observations Not Used	0	


Iteration History	
Iteration	Evaluations	-2 Res Log Like	Criterion	
0	1	189.77268637		
1	2	173.53368467	0.00008793	
2	1	173.53234148	0.00000003	
3	1	173.53234098	0.00000000	


Convergence criteria met.	


Covariance Parameter Estimates	
Cov Parm	Subject	Estimate	
UN(1,1)	Animal	0.8654	
UN(2,1)	Animal	0.1347	
UN(2,2)	Animal	0.3715	
UN(3,1)	Animal	-0.1439	
UN(3,2)	Animal	-0.1518	
UN(3,3)	Animal	0.3539	
UN(4,1)	Animal	-0.2218	
UN(4,2)	Animal	0.008553	
UN(4,3)	Animal	0.08144	
UN(4,4)	Animal	0.3621	


Fit Statistics	
-2 Res Log Likelihood	173.5	
AIC (Smaller is Better)	193.5	
AICC (Smaller is Better)	196.8	
BIC (Smaller is Better)	204.4	


Null Model Likelihood Ratio Test	
DF	Chi-Square	Pr > ChiSq	
9	16.24	0.0620	


Type 3 Tests of Fixed Effects	
Effect	Num DF	Den DF	F Value	Pr > F	
Diet2	1	18	0.39	0.5390	
Group	1	18	0.80	0.3825	
TimeDay	3	18	0.41	0.7479	
Diet2*TimeDay	3	18	1.84	0.1769	
Group*Diet2	1	18	0.28	0.6038	


Least Squares Means	
Effect	Group	Diet2	TimeDay	Estimate	Standard
Error	DF	t Value	Pr > |t|	
Diet2*TimeDay		HFD	0	-0.02889	0.2806	18	-0.10	0.9191	
Diet2*TimeDay		HFD	1	0.1270	0.1839	18	0.69	0.4986	
Diet2*TimeDay		HFD	3	0.4985	0.1795	18	2.78	0.0124	
Diet2*TimeDay		HFD	7	0.6083	0.1816	18	3.35	0.0036	
Diet2*TimeDay		SD	0	0.3771	0.2815	18	1.34	0.1970	
Diet2*TimeDay		SD	1	0.2301	0.1852	18	1.24	0.2301	
Diet2*TimeDay		SD	3	0.3107	0.1809	18	1.72	0.1030	
Diet2*TimeDay		SD	7	-0.03123	0.1829	18	-0.17	0.8664	
Group	IF			0.2081	0.08305	18	2.51	0.0220	
Group	ad_lib			0.3148	0.09098	18	3.46	0.0028	


Differences of Least Squares Means	
Effect	Group	Diet2	TimeDay	_Group	_Diet2	_TimeDay	Estimate	Standard
Error	DF	t Value	Pr > |t|	
Diet2*TimeDay		HFD	0		HFD	1	-0.1559	0.2966	18	-0.53	0.6055	
Diet2*TimeDay		HFD	0		HFD	3	-0.5274	0.3701	18	-1.42	0.1713	
Diet2*TimeDay		HFD	0		HFD	7	-0.6372	0.3898	18	-1.63	0.1195	
Diet2*TimeDay		HFD	0		SD	0	-0.4060	0.3974	18	-1.02	0.3205	
Diet2*TimeDay		HFD	0		SD	1	-0.2590	0.3362	18	-0.77	0.4511	
Diet2*TimeDay		HFD	0		SD	3	-0.3396	0.3338	18	-1.02	0.3225	
Diet2*TimeDay		HFD	0		SD	7	0.002339	0.3350	18	0.01	0.9945	
Diet2*TimeDay		HFD	1		HFD	3	-0.3715	0.3058	18	-1.21	0.2402	
Diet2*TimeDay		HFD	1		HFD	7	-0.4813	0.2552	18	-1.89	0.0756	
Diet2*TimeDay		HFD	1		SD	0	-0.2501	0.3362	18	-0.74	0.4666	
Diet2*TimeDay		HFD	1		SD	1	-0.1031	0.2610	18	-0.39	0.6975	
Diet2*TimeDay		HFD	1		SD	3	-0.1837	0.2580	18	-0.71	0.4856	
Diet2*TimeDay		HFD	1		SD	7	0.1582	0.2594	18	0.61	0.5495	
Diet2*TimeDay		HFD	3		HFD	7	-0.1098	0.2242	18	-0.49	0.6303	
Diet2*TimeDay		HFD	3		SD	0	0.1214	0.3338	18	0.36	0.7204	
Diet2*TimeDay		HFD	3		SD	1	0.2684	0.2580	18	1.04	0.3119	
Diet2*TimeDay		HFD	3		SD	3	0.1878	0.2548	18	0.74	0.4707	
Diet2*TimeDay		HFD	3		SD	7	0.5297	0.2563	18	2.07	0.0535	
Diet2*TimeDay		HFD	7		SD	0	0.2312	0.3350	18	0.69	0.4989	
Diet2*TimeDay		HFD	7		SD	1	0.3782	0.2594	18	1.46	0.1621	
Diet2*TimeDay		HFD	7		SD	3	0.2976	0.2563	18	1.16	0.2608	
Diet2*TimeDay		HFD	7		SD	7	0.6395	0.2578	18	2.48	0.0232	
Diet2*TimeDay		SD	0		SD	1	0.1470	0.2966	18	0.50	0.6261	
Diet2*TimeDay		SD	0		SD	3	0.06642	0.3701	18	0.18	0.8596	
Diet2*TimeDay		SD	0		SD	7	0.4083	0.3898	18	1.05	0.3087	
Diet2*TimeDay		SD	1		SD	3	-0.08058	0.3058	18	-0.26	0.7952	
Diet2*TimeDay		SD	1		SD	7	0.2613	0.2552	18	1.02	0.3194	
Diet2*TimeDay		SD	3		SD	7	0.3419	0.2242	18	1.52	0.1447	
Group	IF			ad_lib			-0.1067	0.1192	18	-0.90	0.3825	

Differences of Least Squares Means	
Effect	Group	Diet2	TimeDay	_Group	_Diet2	_TimeDay	Adjustment	Adj P	
Diet2*TimeDay		HFD	0		HFD	1	Tukey-Kramer	0.9993	
Diet2*TimeDay		HFD	0		HFD	3	Tukey-Kramer	0.8346	
Diet2*TimeDay		HFD	0		HFD	7	Tukey-Kramer	0.7251	
Diet2*TimeDay		HFD	0		SD	0	Tukey-Kramer	0.9648	
Diet2*TimeDay		HFD	0		SD	1	Tukey-Kramer	0.9927	
Diet2*TimeDay		HFD	0		SD	3	Tukey-Kramer	0.9656	
Diet2*TimeDay		HFD	0		SD	7	Tukey-Kramer	1.0000	
Diet2*TimeDay		HFD	1		HFD	3	Tukey-Kramer	0.9172	
Diet2*TimeDay		HFD	1		HFD	7	Tukey-Kramer	0.5761	
Diet2*TimeDay		HFD	1		SD	0	Tukey-Kramer	0.9941	
Diet2*TimeDay		HFD	1		SD	1	Tukey-Kramer	0.9999	
Diet2*TimeDay		HFD	1		SD	3	Tukey-Kramer	0.9955	
Diet2*TimeDay		HFD	1		SD	7	Tukey-Kramer	0.9983	
Diet2*TimeDay		HFD	3		HFD	7	Tukey-Kramer	0.9996	
Diet2*TimeDay		HFD	3		SD	0	Tukey-Kramer	0.9999	
Diet2*TimeDay		HFD	3		SD	1	Tukey-Kramer	0.9613	
Diet2*TimeDay		HFD	3		SD	3	Tukey-Kramer	0.9944	
Diet2*TimeDay		HFD	3		SD	7	Tukey-Kramer	0.4692	
Diet2*TimeDay		HFD	7		SD	0	Tukey-Kramer	0.9962	
Diet2*TimeDay		HFD	7		SD	1	Tukey-Kramer	0.8189	
Diet2*TimeDay		HFD	7		SD	3	Tukey-Kramer	0.9330	
Diet2*TimeDay		HFD	7		SD	7	Tukey-Kramer	0.2636	
Diet2*TimeDay		SD	0		SD	1	Tukey-Kramer	0.9995	
Diet2*TimeDay		SD	0		SD	3	Tukey-Kramer	1.0000	
Diet2*TimeDay		SD	0		SD	7	Tukey-Kramer	0.9599	
Diet2*TimeDay		SD	1		SD	3	Tukey-Kramer	1.0000	
Diet2*TimeDay		SD	1		SD	7	Tukey-Kramer	0.9644	
Diet2*TimeDay		SD	3		SD	7	Tukey-Kramer	0.7852	
Group	IF			ad_lib				.	

Model Information	
Data Set	WORK.DIST	
Dependent Variable	distance	
Covariance Structure	Unstructured	
Subject Effect	Animal	
Estimation Method	REML	
Residual Variance Method	None	
Fixed Effects SE Method	Model-Based	
Degrees of Freedom Method	Between-Within	


Class Level Information	
Class	Levels	Values	
Animal	25	C1 C10 C11 C12 C13 C14 C15 C16 C17 C18 C19 C20 C21 C22 C23 C24 C25 C26 C3 C4 C5 C6 C7 C8 C9	
Group	2	IF ad_lib	
Diet2	2	HFD SD	
distday	8	0 1 2 3 4 5 6 7	


Dimensions	
Covariance Parameters	36	
Columns in X	49	
Columns in Z	0	
Subjects	25	
Max Obs per Subject	8	


Number of Observations	
Number of Observations Read	200	
Number of Observations Used	200	
Number of Observations Not Used	0	


Iteration History	
Iteration	Evaluations	-2 Res Log Like	Criterion	
0	1	529.47200889		
1	4	328.26463301	0.04821770	
2	1	328.08327102	0.00301802	
3	1	328.07310095	0.00001490	
4	1	328.07305279	0.00000000	


Convergence criteria met.	


Covariance Parameter Estimates	
Cov Parm	Subject	Estimate	
UN(1,1)	Animal	0.5123	
UN(2,1)	Animal	0.4274	
UN(2,2)	Animal	0.6480	
UN(3,1)	Animal	0.4710	
UN(3,2)	Animal	0.6345	
UN(3,3)	Animal	1.0720	
UN(4,1)	Animal	0.4236	
UN(4,2)	Animal	0.5666	
UN(4,3)	Animal	0.7372	
UN(4,4)	Animal	1.1780	
UN(5,1)	Animal	0.4983	
UN(5,2)	Animal	0.7223	
UN(5,3)	Animal	0.9356	
UN(5,4)	Animal	0.9611	
UN(5,5)	Animal	1.2697	
UN(6,1)	Animal	0.4477	
UN(6,2)	Animal	0.6562	
UN(6,3)	Animal	0.7204	
UN(6,4)	Animal	0.7856	
UN(6,5)	Animal	0.9171	
UN(6,6)	Animal	0.8927	
UN(7,1)	Animal	0.2530	
UN(7,2)	Animal	0.5423	
UN(7,3)	Animal	0.6648	
UN(7,4)	Animal	0.7132	
UN(7,5)	Animal	0.7587	
UN(7,6)	Animal	0.7283	
UN(7,7)	Animal	0.8523	
UN(8,1)	Animal	0.4653	
UN(8,2)	Animal	0.6510	
UN(8,3)	Animal	0.8570	
UN(8,4)	Animal	0.8064	
UN(8,5)	Animal	0.9512	
UN(8,6)	Animal	0.8484	
UN(8,7)	Animal	0.8207	
UN(8,8)	Animal	1.0964	


Fit Statistics	
-2 Res Log Likelihood	328.1	
AIC (Smaller is Better)	400.1	
AICC (Smaller is Better)	419.4	
BIC (Smaller is Better)	444.0	


Null Model Likelihood Ratio Test	
DF	Chi-Square	Pr > ChiSq	
35	201.40	<.0001	


Type 3 Tests of Fixed Effects	
Effect	Num DF	Den DF	F Value	Pr > F	
Group	1	21	0.72	0.4042	
Diet2	1	21	0.22	0.6413	
Group*Diet2	1	21	0.12	0.7283	
distday	7	21	14.00	<.0001	
Group*distday	7	21	2.65	0.0393	
Diet2*distday	7	21	6.52	0.0004	


Least Squares Means	
Effect	Group	Diet2	distday	Estimate	Standard
Error	DF	t Value	Pr > |t|	
Group*Diet2	IF	HFD		4.0738	0.3188	21	12.78	<.0001	
Group*Diet2	IF	SD		3.8339	0.3188	21	12.02	<.0001	
Group*Diet2	ad_lib	HFD		4.2810	0.3188	21	13.43	<.0001	
Group*Diet2	ad_lib	SD		4.2016	0.2986	21	14.07	<.0001	
Group*distday	IF		0	3.6036	0.2066	21	17.44	<.0001	
Group*distday	IF		1	3.7156	0.2324	21	15.99	<.0001	
Group*distday	IF		2	3.5443	0.2989	21	11.86	<.0001	
Group*distday	IF		3	3.8740	0.3133	21	12.36	<.0001	
Group*distday	IF		4	3.8144	0.3253	21	11.73	<.0001	
Group*distday	IF		5	4.4707	0.2727	21	16.39	<.0001	
Group*distday	IF		6	4.2802	0.2665	21	16.06	<.0001	
Group*distday	IF		7	4.3277	0.3023	21	14.32	<.0001	
Group*distday	ad_lib		0	3.6060	0.1990	21	18.12	<.0001	
Group*distday	ad_lib		1	4.1380	0.2238	21	18.49	<.0001	
Group*distday	ad_lib		2	3.8749	0.2877	21	13.47	<.0001	
Group*distday	ad_lib		3	4.6238	0.3016	21	15.33	<.0001	
Group*distday	ad_lib		4	4.3495	0.3131	21	13.89	<.0001	
Group*distday	ad_lib		5	4.8423	0.2626	21	18.44	<.0001	
Group*distday	ad_lib		6	4.2356	0.2566	21	16.51	<.0001	
Group*distday	ad_lib		7	4.2602	0.2910	21	14.64	<.0001	
Diet2*distday		HFD	0	3.4513	0.2066	21	16.70	<.0001	
Diet2*distday		HFD	1	4.1634	0.2324	21	17.92	<.0001	
Diet2*distday		HFD	2	3.5209	0.2989	21	11.78	<.0001	
Diet2*distday		HFD	3	4.6474	0.3133	21	14.83	<.0001	
Diet2*distday		HFD	4	4.1954	0.3253	21	12.90	<.0001	
Diet2*distday		HFD	5	4.7978	0.2727	21	17.59	<.0001	
Diet2*distday		HFD	6	4.2446	0.2665	21	15.93	<.0001	
Diet2*distday		HFD	7	4.3982	0.3023	21	14.55	<.0001	
Diet2*distday		SD	0	3.7584	0.1990	21	18.89	<.0001	
Diet2*distday		SD	1	3.6902	0.2238	21	16.49	<.0001	
Diet2*distday		SD	2	3.8984	0.2877	21	13.55	<.0001	
Diet2*distday		SD	3	3.8504	0.3016	21	12.77	<.0001	
Diet2*distday		SD	4	3.9685	0.3131	21	12.67	<.0001	
Diet2*distday		SD	5	4.5151	0.2626	21	17.19	<.0001	
Diet2*distday		SD	6	4.2712	0.2566	21	16.65	<.0001	
Diet2*distday		SD	7	4.1897	0.2910	21	14.40	<.0001	


Differences of Least Squares Means	
Effect	Group	Diet2	distday	_Group	_Diet2	_distday	Estimate	Standard
Error	DF	t Value	Pr > |t|	
Group*Diet2	IF	HFD		IF	SD		0.2399	0.4122	21	0.58	0.5667	
Group*Diet2	IF	HFD		ad_lib	HFD		-0.2072	0.4122	21	-0.50	0.6205	
Group*Diet2	IF	HFD		ad_lib	SD		-0.1278	0.4685	21	-0.27	0.7876	
Group*Diet2	IF	SD		ad_lib	HFD		-0.4471	0.4865	21	-0.92	0.3686	
Group*Diet2	IF	SD		ad_lib	SD		-0.3678	0.4028	21	-0.91	0.3715	
Group*Diet2	ad_lib	HFD		ad_lib	SD		0.07932	0.4028	21	0.20	0.8458	
Group*distday	IF		0	IF		1	-0.1120	0.1596	21	-0.70	0.4905	
Group*distday	IF		0	IF		2	0.05928	0.2314	21	0.26	0.8003	
Group*distday	IF		0	IF		3	-0.2704	0.2651	21	-1.02	0.3193	
Group*distday	IF		0	IF		4	-0.2108	0.2558	21	-0.82	0.4191	
Group*distday	IF		0	IF		5	-0.8671	0.2061	21	-4.21	0.0004	
Group*distday	IF		0	IF		6	-0.6766	0.2675	21	-2.53	0.0195	
Group*distday	IF		0	IF		7	-0.7241	0.2377	21	-3.05	0.0061	
Group*distday	IF		0	ad_lib		0	-0.00244	0.2869	21	-0.01	0.9933	
Group*distday	IF		0	ad_lib		1	-0.5344	0.3046	21	-1.75	0.0939	
Group*distday	IF		0	ad_lib		2	-0.2713	0.3542	21	-0.77	0.4523	
Group*distday	IF		0	ad_lib		3	-1.0202	0.3656	21	-2.79	0.0110	
Group*distday	IF		0	ad_lib		4	-0.7459	0.3751	21	-1.99	0.0600	
Group*distday	IF		0	ad_lib		5	-1.2387	0.3341	21	-3.71	0.0013	
Group*distday	IF		0	ad_lib		6	-0.6320	0.3294	21	-1.92	0.0688	
Group*distday	IF		0	ad_lib		7	-0.6566	0.3569	21	-1.84	0.0800	
Group*distday	IF		1	IF		2	0.1713	0.1939	21	0.88	0.3870	
Group*distday	IF		1	IF		3	-0.1584	0.2403	21	-0.66	0.5170	
Group*distday	IF		1	IF		4	-0.09882	0.1986	21	-0.50	0.6239	
Group*distday	IF		1	IF		5	-0.7551	0.1379	21	-5.47	<.0001	
Group*distday	IF		1	IF		6	-0.5646	0.1861	21	-3.03	0.0063	
Group*distday	IF		1	IF		7	-0.6121	0.1920	21	-3.19	0.0044	
Group*distday	IF		1	ad_lib		0	0.1096	0.3059	21	0.36	0.7238	
Group*distday	IF		1	ad_lib		1	-0.4224	0.3226	21	-1.31	0.2046	
Group*distday	IF		1	ad_lib		2	-0.1593	0.3698	21	-0.43	0.6711	
Group*distday	IF		1	ad_lib		3	-0.9082	0.3807	21	-2.39	0.0266	
Group*distday	IF		1	ad_lib		4	-0.6339	0.3899	21	-1.63	0.1189	
Group*distday	IF		1	ad_lib		5	-1.1267	0.3506	21	-3.21	0.0042	
Group*distday	IF		1	ad_lib		6	-0.5200	0.3462	21	-1.50	0.1480	
Group*distday	IF		1	ad_lib		7	-0.5446	0.3724	21	-1.46	0.1584	
Group*distday	IF		2	IF		3	-0.3297	0.2542	21	-1.30	0.2088	
Group*distday	IF		2	IF		4	-0.2701	0.1980	21	-1.36	0.1869	
Group*distday	IF		2	IF		5	-0.9263	0.2089	21	-4.43	0.0002	
Group*distday	IF		2	IF		6	-0.7359	0.2226	21	-3.31	0.0034	
Group*distday	IF		2	IF		7	-0.7834	0.1946	21	-4.03	0.0006	
Group*distday	IF		2	ad_lib		0	-0.06172	0.3591	21	-0.17	0.8652	
Group*distday	IF		2	ad_lib		1	-0.5937	0.3734	21	-1.59	0.1268	
Group*distday	IF		2	ad_lib		2	-0.3306	0.4149	21	-0.80	0.4345	
Group*distday	IF		2	ad_lib		3	-1.0795	0.4246	21	-2.54	0.0190	
Group*distday	IF		2	ad_lib		4	-0.8052	0.4329	21	-1.86	0.0769	
Group*distday	IF		2	ad_lib		5	-1.2980	0.3979	21	-3.26	0.0037	
Group*distday	IF		2	ad_lib		6	-0.6913	0.3939	21	-1.75	0.0939	
Group*distday	IF		2	ad_lib		7	-0.7159	0.4171	21	-1.72	0.1008	
Group*distday	IF		3	IF		4	0.05955	0.2093	21	0.28	0.7787	
Group*distday	IF		3	IF		5	-0.5967	0.2040	21	-2.92	0.0081	
Group*distday	IF		3	IF		6	-0.4062	0.2243	21	-1.81	0.0845	
Group*distday	IF		3	IF		7	-0.4537	0.2348	21	-1.93	0.0669	
Group*distday	IF		3	ad_lib		0	0.2679	0.3712	21	0.72	0.4783	
Group*distday	IF		3	ad_lib		1	-0.2640	0.3850	21	-0.69	0.5004	
Group*distday	IF		3	ad_lib		2	-0.00092	0.4254	21	-0.00	0.9983	
Group*distday	IF		3	ad_lib		3	-0.7498	0.4349	21	-1.72	0.0994	
Group*distday	IF		3	ad_lib		4	-0.4755	0.4430	21	-1.07	0.2952	
Group*distday	IF		3	ad_lib		5	-0.9683	0.4088	21	-2.37	0.0275	
Group*distday	IF		3	ad_lib		6	-0.3616	0.4050	21	-0.89	0.3820	
Group*distday	IF		3	ad_lib		7	-0.3862	0.4276	21	-0.90	0.3766	
Group*distday	IF		4	IF		5	-0.6562	0.1654	21	-3.97	0.0007	
Group*distday	IF		4	IF		6	-0.4658	0.2245	21	-2.08	0.0505	
Group*distday	IF		4	IF		7	-0.5133	0.1966	21	-2.61	0.0163	
Group*distday	IF		4	ad_lib		0	0.2084	0.3813	21	0.55	0.5905	
Group*distday	IF		4	ad_lib		1	-0.3236	0.3948	21	-0.82	0.4217	
Group*distday	IF		4	ad_lib		2	-0.06047	0.4343	21	-0.14	0.8906	
Group*distday	IF		4	ad_lib		3	-0.8094	0.4436	21	-1.82	0.0823	
Group*distday	IF		4	ad_lib		4	-0.5351	0.4515	21	-1.19	0.2492	
Group*distday	IF		4	ad_lib		5	-1.0279	0.4180	21	-2.46	0.0227	
Group*distday	IF		4	ad_lib		6	-0.4212	0.4143	21	-1.02	0.3209	
Group*distday	IF		4	ad_lib		7	-0.4458	0.4364	21	-1.02	0.3187	
Group*distday	IF		5	IF		6	0.1904	0.1550	21	1.23	0.2328	
Group*distday	IF		5	IF		7	0.1429	0.1561	21	0.92	0.3701	
Group*distday	IF		5	ad_lib		0	0.8646	0.3376	21	2.56	0.0182	
Group*distday	IF		5	ad_lib		1	0.3327	0.3528	21	0.94	0.3564	
Group*distday	IF		5	ad_lib		2	0.5958	0.3965	21	1.50	0.1478	
Group*distday	IF		5	ad_lib		3	-0.1531	0.4066	21	-0.38	0.7103	
Group*distday	IF		5	ad_lib		4	0.1212	0.4153	21	0.29	0.7733	
Group*distday	IF		5	ad_lib		5	-0.3716	0.3786	21	-0.98	0.3375	
Group*distday	IF		5	ad_lib		6	0.2351	0.3745	21	0.63	0.5369	
Group*distday	IF		5	ad_lib		7	0.2104	0.3988	21	0.53	0.6033	
Group*distday	IF		6	IF		7	-0.04750	0.1600	21	-0.30	0.7695	
Group*distday	IF		6	ad_lib		0	0.6742	0.3326	21	2.03	0.0555	
Group*distday	IF		6	ad_lib		1	0.1422	0.3480	21	0.41	0.6869	
Group*distday	IF		6	ad_lib		2	0.4053	0.3922	21	1.03	0.3131	
Group*distday	IF		6	ad_lib		3	-0.3436	0.4025	21	-0.85	0.4029	
Group*distday	IF		6	ad_lib		4	-0.06926	0.4112	21	-0.17	0.8678	
Group*distday	IF		6	ad_lib		5	-0.5621	0.3741	21	-1.50	0.1479	
Group*distday	IF		6	ad_lib		6	0.04463	0.3699	21	0.12	0.9051	
Group*distday	IF		6	ad_lib		7	0.02000	0.3946	21	0.05	0.9601	
Group*distday	IF		7	ad_lib		0	0.7217	0.3619	21	1.99	0.0593	
Group*distday	IF		7	ad_lib		1	0.1897	0.3761	21	0.50	0.6192	
Group*distday	IF		7	ad_lib		2	0.4528	0.4173	21	1.09	0.2902	
Group*distday	IF		7	ad_lib		3	-0.2961	0.4270	21	-0.69	0.4957	
Group*distday	IF		7	ad_lib		4	-0.02176	0.4352	21	-0.05	0.9606	
Group*distday	IF		7	ad_lib		5	-0.5146	0.4004	21	-1.29	0.2127	
Group*distday	IF		7	ad_lib		6	0.09213	0.3965	21	0.23	0.8185	
Group*distday	IF		7	ad_lib		7	0.06750	0.4196	21	0.16	0.8737	
Group*distday	ad_lib		0	ad_lib		1	-0.5319	0.1536	21	-3.46	0.0023	
Group*distday	ad_lib		0	ad_lib		2	-0.2689	0.2226	21	-1.21	0.2406	
Group*distday	ad_lib		0	ad_lib		3	-1.0178	0.2551	21	-3.99	0.0007	
Group*distday	ad_lib		0	ad_lib		4	-0.7434	0.2462	21	-3.02	0.0065	
Group*distday	ad_lib		0	ad_lib		5	-1.2362	0.1983	21	-6.23	<.0001	
Group*distday	ad_lib		0	ad_lib		6	-0.6295	0.2574	21	-2.45	0.0233	
Group*distday	ad_lib		0	ad_lib		7	-0.6542	0.2288	21	-2.86	0.0094	
Group*distday	ad_lib		1	ad_lib		2	0.2631	0.1866	21	1.41	0.1731	
Group*distday	ad_lib		1	ad_lib		3	-0.4858	0.2312	21	-2.10	0.0479	
Group*distday	ad_lib		1	ad_lib		4	-0.2115	0.1911	21	-1.11	0.2808	
Group*distday	ad_lib		1	ad_lib		5	-0.7043	0.1327	21	-5.31	<.0001	
Group*distday	ad_lib		1	ad_lib		6	-0.09761	0.1791	21	-0.55	0.5915	
Group*distday	ad_lib		1	ad_lib		7	-0.1222	0.1847	21	-0.66	0.5154	
Group*distday	ad_lib		2	ad_lib		3	-0.7489	0.2446	21	-3.06	0.0059	
Group*distday	ad_lib		2	ad_lib		4	-0.4746	0.1905	21	-2.49	0.0212	
Group*distday	ad_lib		2	ad_lib		5	-0.9674	0.2010	21	-4.81	<.0001	
Group*distday	ad_lib		2	ad_lib		6	-0.3607	0.2142	21	-1.68	0.1070	
Group*distday	ad_lib		2	ad_lib		7	-0.3853	0.1872	21	-2.06	0.0522	
Group*distday	ad_lib		3	ad_lib		4	0.2743	0.2014	21	1.36	0.1875	
Group*distday	ad_lib		3	ad_lib		5	-0.2185	0.1963	21	-1.11	0.2783	
Group*distday	ad_lib		3	ad_lib		6	0.3882	0.2159	21	1.80	0.0865	
Group*distday	ad_lib		3	ad_lib		7	0.3636	0.2259	21	1.61	0.1225	
Group*distday	ad_lib		4	ad_lib		5	-0.4928	0.1591	21	-3.10	0.0055	
Group*distday	ad_lib		4	ad_lib		6	0.1139	0.2160	21	0.53	0.6035	
Group*distday	ad_lib		4	ad_lib		7	0.08926	0.1892	21	0.47	0.6419	
Group*distday	ad_lib		5	ad_lib		6	0.6067	0.1491	21	4.07	0.0006	
Group*distday	ad_lib		5	ad_lib		7	0.5821	0.1502	21	3.88	0.0009	
Group*distday	ad_lib		6	ad_lib		7	-0.02463	0.1540	21	-0.16	0.8744	
Diet2*distday		HFD	0		HFD	1	-0.7121	0.1596	21	-4.46	0.0002	
Diet2*distday		HFD	0		HFD	2	-0.06958	0.2314	21	-0.30	0.7666	
Diet2*distday		HFD	0		HFD	3	-1.1961	0.2651	21	-4.51	0.0002	
Diet2*distday		HFD	0		HFD	4	-0.7441	0.2558	21	-2.91	0.0084	
Diet2*distday		HFD	0		HFD	5	-1.3466	0.2061	21	-6.53	<.0001	
Diet2*distday		HFD	0		HFD	6	-0.7933	0.2675	21	-2.97	0.0074	
Diet2*distday		HFD	0		HFD	7	-0.9469	0.2377	21	-3.98	0.0007	
Diet2*distday		HFD	0		SD	0	-0.3071	0.2869	21	-1.07	0.2965	
Diet2*distday		HFD	0		SD	1	-0.2389	0.3046	21	-0.78	0.4415	
Diet2*distday		HFD	0		SD	2	-0.4471	0.3542	21	-1.26	0.2207	
Diet2*distday		HFD	0		SD	3	-0.3991	0.3656	21	-1.09	0.2873	
Diet2*distday		HFD	0		SD	4	-0.5172	0.3751	21	-1.38	0.1825	
Diet2*distday		HFD	0		SD	5	-1.0639	0.3341	21	-3.18	0.0045	
Diet2*distday		HFD	0		SD	6	-0.8200	0.3294	21	-2.49	0.0213	
Diet2*distday		HFD	0		SD	7	-0.7385	0.3569	21	-2.07	0.0511	
Diet2*distday		HFD	1		HFD	2	0.6425	0.1939	21	3.31	0.0033	
Diet2*distday		HFD	1		HFD	3	-0.4840	0.2403	21	-2.01	0.0570	
Diet2*distday		HFD	1		HFD	4	-0.03203	0.1986	21	-0.16	0.8734	
Diet2*distday		HFD	1		HFD	5	-0.6344	0.1379	21	-4.60	0.0002	
Diet2*distday		HFD	1		HFD	6	-0.08122	0.1861	21	-0.44	0.6670	
Diet2*distday		HFD	1		HFD	7	-0.2348	0.1920	21	-1.22	0.2348	
Diet2*distday		HFD	1		SD	0	0.4050	0.3059	21	1.32	0.1998	
Diet2*distday		HFD	1		SD	1	0.4732	0.3226	21	1.47	0.1573	
Diet2*distday		HFD	1		SD	2	0.2650	0.3698	21	0.72	0.4816	
Diet2*distday		HFD	1		SD	3	0.3130	0.3807	21	0.82	0.4203	
Diet2*distday		HFD	1		SD	4	0.1949	0.3899	21	0.50	0.6224	
Diet2*distday		HFD	1		SD	5	-0.3517	0.3506	21	-1.00	0.3272	
Diet2*distday		HFD	1		SD	6	-0.1078	0.3462	21	-0.31	0.7585	
Diet2*distday		HFD	1		SD	7	-0.02635	0.3724	21	-0.07	0.9442	
Diet2*distday		HFD	2		HFD	3	-1.1265	0.2542	21	-4.43	0.0002	
Diet2*distday		HFD	2		HFD	4	-0.6746	0.1980	21	-3.41	0.0027	
Diet2*distday		HFD	2		HFD	5	-1.2770	0.2089	21	-6.11	<.0001	
Diet2*distday		HFD	2		HFD	6	-0.7238	0.2226	21	-3.25	0.0038	
Diet2*distday		HFD	2		HFD	7	-0.8774	0.1946	21	-4.51	0.0002	
Diet2*distday		HFD	2		SD	0	-0.2375	0.3591	21	-0.66	0.5155	
Diet2*distday		HFD	2		SD	1	-0.1694	0.3734	21	-0.45	0.6548	
Diet2*distday		HFD	2		SD	2	-0.3775	0.4149	21	-0.91	0.3732	
Diet2*distday		HFD	2		SD	3	-0.3295	0.4246	21	-0.78	0.4464	
Diet2*distday		HFD	2		SD	4	-0.4477	0.4329	21	-1.03	0.3128	
Diet2*distday		HFD	2		SD	5	-0.9943	0.3979	21	-2.50	0.0208	
Diet2*distday		HFD	2		SD	6	-0.7504	0.3939	21	-1.90	0.0706	
Diet2*distday		HFD	2		SD	7	-0.6689	0.4171	21	-1.60	0.1238	
Diet2*distday		HFD	3		HFD	4	0.4520	0.2093	21	2.16	0.0425	
Diet2*distday		HFD	3		HFD	5	-0.1504	0.2040	21	-0.74	0.4690	
Diet2*distday		HFD	3		HFD	6	0.4028	0.2243	21	1.80	0.0870	
Diet2*distday		HFD	3		HFD	7	0.2492	0.2348	21	1.06	0.3006	
Diet2*distday		HFD	3		SD	0	0.8890	0.3712	21	2.40	0.0260	
Diet2*distday		HFD	3		SD	1	0.9572	0.3850	21	2.49	0.0214	
Diet2*distday		HFD	3		SD	2	0.7490	0.4254	21	1.76	0.0928	
Diet2*distday		HFD	3		SD	3	0.7970	0.4349	21	1.83	0.0811	
Diet2*distday		HFD	3		SD	4	0.6789	0.4430	21	1.53	0.1403	
Diet2*distday		HFD	3		SD	5	0.1323	0.4088	21	0.32	0.7495	
Diet2*distday		HFD	3		SD	6	0.3762	0.4050	21	0.93	0.3635	
Diet2*distday		HFD	3		SD	7	0.4577	0.4276	21	1.07	0.2966	
Diet2*distday		HFD	4		HFD	5	-0.6024	0.1654	21	-3.64	0.0015	
Diet2*distday		HFD	4		HFD	6	-0.04918	0.2245	21	-0.22	0.8287	
Diet2*distday		HFD	4		HFD	7	-0.2028	0.1966	21	-1.03	0.3140	
Diet2*distday		HFD	4		SD	0	0.4370	0.3813	21	1.15	0.2647	
Diet2*distday		HFD	4		SD	1	0.5052	0.3948	21	1.28	0.2146	
Diet2*distday		HFD	4		SD	2	0.2970	0.4343	21	0.68	0.5015	
Diet2*distday		HFD	4		SD	3	0.3450	0.4436	21	0.78	0.4454	
Diet2*distday		HFD	4		SD	4	0.2269	0.4515	21	0.50	0.6205	
Diet2*distday		HFD	4		SD	5	-0.3197	0.4180	21	-0.76	0.4529	
Diet2*distday		HFD	4		SD	6	-0.07580	0.4143	21	-0.18	0.8566	
Diet2*distday		HFD	4		SD	7	0.005678	0.4364	21	0.01	0.9897	
Diet2*distday		HFD	5		HFD	6	0.5532	0.1550	21	3.57	0.0018	
Diet2*distday		HFD	5		HFD	7	0.3996	0.1561	21	2.56	0.0182	
Diet2*distday		HFD	5		SD	0	1.0394	0.3376	21	3.08	0.0057	
Diet2*distday		HFD	5		SD	1	1.1076	0.3528	21	3.14	0.0050	
Diet2*distday		HFD	5		SD	2	0.8995	0.3965	21	2.27	0.0339	
Diet2*distday		HFD	5		SD	3	0.9474	0.4066	21	2.33	0.0299	
Diet2*distday		HFD	5		SD	4	0.8293	0.4153	21	2.00	0.0589	
Diet2*distday		HFD	5		SD	5	0.2827	0.3786	21	0.75	0.4635	
Diet2*distday		HFD	5		SD	6	0.5266	0.3745	21	1.41	0.1743	
Diet2*distday		HFD	5		SD	7	0.6081	0.3988	21	1.52	0.1423	
Diet2*distday		HFD	6		HFD	7	-0.1536	0.1600	21	-0.96	0.3480	
Diet2*distday		HFD	6		SD	0	0.4862	0.3326	21	1.46	0.1586	
Diet2*distday		HFD	6		SD	1	0.5544	0.3480	21	1.59	0.1261	
Diet2*distday		HFD	6		SD	2	0.3462	0.3922	21	0.88	0.3873	
Diet2*distday		HFD	6		SD	3	0.3942	0.4025	21	0.98	0.3385	
Diet2*distday		HFD	6		SD	4	0.2761	0.4112	21	0.67	0.5092	
Diet2*distday		HFD	6		SD	5	-0.2705	0.3741	21	-0.72	0.4776	
Diet2*distday		HFD	6		SD	6	-0.02662	0.3699	21	-0.07	0.9433	
Diet2*distday		HFD	6		SD	7	0.05486	0.3946	21	0.14	0.8907	
Diet2*distday		HFD	7		SD	0	0.6398	0.3619	21	1.77	0.0916	
Diet2*distday		HFD	7		SD	1	0.7080	0.3761	21	1.88	0.0737	
Diet2*distday		HFD	7		SD	2	0.4998	0.4173	21	1.20	0.2444	
Diet2*distday		HFD	7		SD	3	0.5478	0.4270	21	1.28	0.2135	
Diet2*distday		HFD	7		SD	4	0.4297	0.4352	21	0.99	0.3347	
Diet2*distday		HFD	7		SD	5	-0.1169	0.4004	21	-0.29	0.7732	
Diet2*distday		HFD	7		SD	6	0.1270	0.3965	21	0.32	0.7519	
Diet2*distday		HFD	7		SD	7	0.2085	0.4196	21	0.50	0.6244	
Diet2*distday		SD	0		SD	1	0.06817	0.1536	21	0.44	0.6616	
Diet2*distday		SD	0		SD	2	-0.1400	0.2226	21	-0.63	0.5363	
Diet2*distday		SD	0		SD	3	-0.09200	0.2551	21	-0.36	0.7219	
Diet2*distday		SD	0		SD	4	-0.2101	0.2462	21	-0.85	0.4030	
Diet2*distday		SD	0		SD	5	-0.7567	0.1983	21	-3.82	0.0010	
Diet2*distday		SD	0		SD	6	-0.5128	0.2574	21	-1.99	0.0595	
Diet2*distday		SD	0		SD	7	-0.4314	0.2288	21	-1.89	0.0733	
Diet2*distday		SD	1		SD	2	-0.2082	0.1866	21	-1.12	0.2771	
Diet2*distday		SD	1		SD	3	-0.1602	0.2312	21	-0.69	0.4960	
Diet2*distday		SD	1		SD	4	-0.2783	0.1911	21	-1.46	0.1600	
Diet2*distday		SD	1		SD	5	-0.8249	0.1327	21	-6.22	<.0001	
Diet2*distday		SD	1		SD	6	-0.5810	0.1791	21	-3.24	0.0039	
Diet2*distday		SD	1		SD	7	-0.4995	0.1847	21	-2.70	0.0133	
Diet2*distday		SD	2		SD	3	0.04799	0.2446	21	0.20	0.8463	
Diet2*distday		SD	2		SD	4	-0.07012	0.1905	21	-0.37	0.7165	
Diet2*distday		SD	2		SD	5	-0.6167	0.2010	21	-3.07	0.0058	
Diet2*distday		SD	2		SD	6	-0.3728	0.2142	21	-1.74	0.0964	
Diet2*distday		SD	2		SD	7	-0.2914	0.1872	21	-1.56	0.1346	
Diet2*distday		SD	3		SD	4	-0.1181	0.2014	21	-0.59	0.5637	
Diet2*distday		SD	3		SD	5	-0.6647	0.1963	21	-3.39	0.0028	
Diet2*distday		SD	3		SD	6	-0.4208	0.2159	21	-1.95	0.0647	
Diet2*distday		SD	3		SD	7	-0.3394	0.2259	21	-1.50	0.1480	
Diet2*distday		SD	4		SD	5	-0.5466	0.1591	21	-3.44	0.0025	
Diet2*distday		SD	4		SD	6	-0.3027	0.2160	21	-1.40	0.1757	
Diet2*distday		SD	4		SD	7	-0.2212	0.1892	21	-1.17	0.2553	
Diet2*distday		SD	5		SD	6	0.2439	0.1491	21	1.64	0.1169	
Diet2*distday		SD	5		SD	7	0.3254	0.1502	21	2.17	0.0419	
Diet2*distday		SD	6		SD	7	0.08148	0.1540	21	0.53	0.6022	

Model Information	
Data Set	WORK.DIST	
Dependent Variable	distance	
Covariance Structure	Unstructured	
Subject Effect	Animal	
Estimation Method	REML	
Residual Variance Method	None	
Fixed Effects SE Method	Model-Based	
Degrees of Freedom Method	Between-Within	


Class Level Information	
Class	Levels	Values	
Animal	22	T1 T10 T11 T12 T13 T14 T15 T17 T2 T21 T22 T23 T24 T26 T27 T3 T4 T5 T6 T7 T8 T9	
Group	2	IF ad_lib	
Diet2	2	HFD SD	
distday	8	0 1 2 3 4 5 6 7	


Dimensions	
Covariance Parameters	36	
Columns in X	49	
Columns in Z	0	
Subjects	22	
Max Obs per Subject	8	


Number of Observations	
Number of Observations Read	175	
Number of Observations Used	175	
Number of Observations Not Used	0	


Iteration History	
Iteration	Evaluations	-2 Res Log Like	Criterion	
0	1	358.44015106		
1	2	259.43803357	0.00844640	
2	1	259.36408285	0.00015468	
3	1	259.36280204	0.00000008	
4	1	259.36280137	0.00000000	


Convergence criteria met.	


Covariance Parameter Estimates	
Cov Parm	Subject	Estimate	
UN(1,1)	Animal	0.6690	
UN(2,1)	Animal	0.1407	
UN(2,2)	Animal	0.5068	
UN(3,1)	Animal	0.1462	
UN(3,2)	Animal	0.1450	
UN(3,3)	Animal	0.2817	
UN(4,1)	Animal	0.2239	
UN(4,2)	Animal	0.2078	
UN(4,3)	Animal	0.2669	
UN(4,4)	Animal	0.4435	
UN(5,1)	Animal	0.1811	
UN(5,2)	Animal	0.3651	
UN(5,3)	Animal	0.1747	
UN(5,4)	Animal	0.3189	
UN(5,5)	Animal	0.4912	
UN(6,1)	Animal	-0.00068	
UN(6,2)	Animal	0.06686	
UN(6,3)	Animal	0.1785	
UN(6,4)	Animal	0.2001	
UN(6,5)	Animal	0.1191	
UN(6,6)	Animal	0.4536	
UN(7,1)	Animal	0.2000	
UN(7,2)	Animal	0.1600	
UN(7,3)	Animal	0.1518	
UN(7,4)	Animal	0.2779	
UN(7,5)	Animal	0.2632	
UN(7,6)	Animal	0.2122	
UN(7,7)	Animal	0.3773	
UN(8,1)	Animal	0.1043	
UN(8,2)	Animal	-0.1304	
UN(8,3)	Animal	0.04891	
UN(8,4)	Animal	0.1515	
UN(8,5)	Animal	0.04697	
UN(8,6)	Animal	0.2223	
UN(8,7)	Animal	0.1543	
UN(8,8)	Animal	0.3661	


Fit Statistics	
-2 Res Log Likelihood	259.4	
AIC (Smaller is Better)	331.4	
AICC (Smaller is Better)	354.9	
BIC (Smaller is Better)	370.6	


Null Model Likelihood Ratio Test	
DF	Chi-Square	Pr > ChiSq	
35	99.08	<.0001	


Type 3 Tests of Fixed Effects	
Effect	Num DF	Den DF	F Value	Pr > F	
Group	1	18	0.11	0.7474	
Diet2	1	18	0.41	0.5304	
Group*Diet2	1	18	9.89	0.0056	
distday	7	18	5.32	0.0020	
Group*distday	7	18	1.21	0.3466	
Diet2*distday	7	18	0.92	0.5112	


Least Squares Means	
Effect	Group	Diet2	distday	Estimate	Standard
Error	DF	t Value	Pr > |t|	
Group*Diet2	IF	HFD		3.3696	0.1864	18	18.08	<.0001	
Group*Diet2	IF	SD		3.9346	0.1609	18	24.46	<.0001	
Group*Diet2	ad_lib	HFD		3.7471	0.1723	18	21.75	<.0001	
Group*Diet2	ad_lib	SD		3.4301	0.2043	18	16.79	<.0001	
Group*distday	IF		0	3.1704	0.2380	18	13.32	<.0001	
Group*distday	IF		1	3.1867	0.2074	18	15.37	<.0001	
Group*distday	IF		2	3.7592	0.1547	18	24.29	<.0001	
Group*distday	IF		3	3.6461	0.1940	18	18.79	<.0001	
Group*distday	IF		4	3.6592	0.2042	18	17.92	<.0001	
Group*distday	IF		5	3.8119	0.1962	18	19.42	<.0001	
Group*distday	IF		6	4.0315	0.1790	18	22.52	<.0001	
Group*distday	IF		7	3.9516	0.1763	18	22.41	<.0001	
Group*distday	ad_lib		0	3.6134	0.2345	18	15.41	<.0001	
Group*distday	ad_lib		1	3.3293	0.2221	18	14.99	<.0001	
Group*distday	ad_lib		2	3.4677	0.1637	18	21.18	<.0001	
Group*distday	ad_lib		3	3.6206	0.2057	18	17.60	<.0001	
Group*distday	ad_lib		4	3.7664	0.2264	18	16.64	<.0001	
Group*distday	ad_lib		5	3.2927	0.2202	18	14.95	<.0001	
Group*distday	ad_lib		6	3.8148	0.2024	18	18.84	<.0001	
Group*distday	ad_lib		7	3.8039	0.1989	18	19.12	<.0001	
Diet2*distday		HFD	0	3.3977	0.2472	18	13.75	<.0001	
Diet2*distday		HFD	1	3.1289	0.2152	18	14.54	<.0001	
Diet2*distday		HFD	2	3.5079	0.1605	18	21.86	<.0001	
Diet2*distday		HFD	3	3.5050	0.2013	18	17.41	<.0001	
Diet2*distday		HFD	4	3.4694	0.2119	18	16.38	<.0001	
Diet2*distday		HFD	5	3.5823	0.2036	18	17.59	<.0001	
Diet2*distday		HFD	6	3.9772	0.1857	18	21.42	<.0001	
Diet2*distday		HFD	7	3.8984	0.1829	18	21.31	<.0001	
Diet2*distday		SD	0	3.3861	0.2261	18	14.98	<.0001	
Diet2*distday		SD	1	3.3871	0.2142	18	15.81	<.0001	
Diet2*distday		SD	2	3.7189	0.1580	18	23.53	<.0001	
Diet2*distday		SD	3	3.7617	0.1983	18	18.97	<.0001	
Diet2*distday		SD	4	3.9562	0.2183	18	18.12	<.0001	
Diet2*distday		SD	5	3.5223	0.2125	18	16.57	<.0001	
Diet2*distday		SD	6	3.8691	0.1954	18	19.80	<.0001	
Diet2*distday		SD	7	3.8571	0.1922	18	20.06	<.0001	


Differences of Least Squares Means	
Effect	Group	Diet2	distday	_Group	_Diet2	_distday	Estimate	Standard
Error	DF	t Value	Pr > |t|	
Group*Diet2	IF	HFD		IF	SD		-0.5650	0.2306	18	-2.45	0.0248	
Group*Diet2	IF	HFD		ad_lib	HFD		-0.3775	0.2361	18	-1.60	0.1272	
Group*Diet2	IF	HFD		ad_lib	SD		-0.06051	0.2979	18	-0.20	0.8413	
Group*Diet2	IF	SD		ad_lib	HFD		0.1874	0.2485	18	0.75	0.4604	
Group*Diet2	IF	SD		ad_lib	SD		0.5045	0.2427	18	2.08	0.0522	
Group*Diet2	ad_lib	HFD		ad_lib	SD		0.3170	0.2475	18	1.28	0.2165	
Group*distday	IF		0	IF		1	-0.01632	0.2748	18	-0.06	0.9533	
Group*distday	IF		0	IF		2	-0.5888	0.2357	18	-2.50	0.0224	
Group*distday	IF		0	IF		3	-0.4757	0.2369	18	-2.01	0.0599	
Group*distday	IF		0	IF		4	-0.4889	0.2596	18	-1.88	0.0760	
Group*distday	IF		0	IF		5	-0.6416	0.3082	18	-2.08	0.0519	
Group*distday	IF		0	IF		6	-0.8612	0.2337	18	-3.68	0.0017	
Group*distday	IF		0	IF		7	-0.7813	0.2644	18	-2.95	0.0085	
Group*distday	IF		0	ad_lib		0	-0.4430	0.3333	18	-1.33	0.2004	
Group*distday	IF		0	ad_lib		1	-0.1589	0.3267	18	-0.49	0.6325	
Group*distday	IF		0	ad_lib		2	-0.2973	0.2901	18	-1.02	0.3189	
Group*distday	IF		0	ad_lib		3	-0.4503	0.3160	18	-1.43	0.1712	
Group*distday	IF		0	ad_lib		4	-0.5960	0.3296	18	-1.81	0.0873	
Group*distday	IF		0	ad_lib		5	-0.1223	0.3248	18	-0.38	0.7109	
Group*distday	IF		0	ad_lib		6	-0.6445	0.3128	18	-2.06	0.0541	
Group*distday	IF		0	ad_lib		7	-0.6336	0.3092	18	-2.05	0.0553	
Group*distday	IF		1	IF		2	-0.5725	0.2054	18	-2.79	0.0122	
Group*distday	IF		1	IF		3	-0.4594	0.2127	18	-2.16	0.0445	
Group*distday	IF		1	IF		4	-0.4726	0.1506	18	-3.14	0.0057	
Group*distday	IF		1	IF		5	-0.6252	0.2645	18	-2.36	0.0296	
Group*distday	IF		1	IF		6	-0.8449	0.2185	18	-3.87	0.0011	
Group*distday	IF		1	IF		7	-0.7649	0.3097	18	-2.47	0.0238	
Group*distday	IF		1	ad_lib		0	-0.4267	0.3141	18	-1.36	0.1911	
Group*distday	IF		1	ad_lib		1	-0.1426	0.3053	18	-0.47	0.6460	
Group*distday	IF		1	ad_lib		2	-0.2810	0.2643	18	-1.06	0.3016	
Group*distday	IF		1	ad_lib		3	-0.4340	0.2927	18	-1.48	0.1555	
Group*distday	IF		1	ad_lib		4	-0.5797	0.3081	18	-1.88	0.0761	
Group*distday	IF		1	ad_lib		5	-0.1060	0.3019	18	-0.35	0.7296	
Group*distday	IF		1	ad_lib		6	-0.6282	0.2901	18	-2.17	0.0440	
Group*distday	IF		1	ad_lib		7	-0.6173	0.2861	18	-2.16	0.0447	
Group*distday	IF		2	IF		3	0.1131	0.1273	18	0.89	0.3863	
Group*distday	IF		2	IF		4	0.09993	0.1894	18	0.53	0.6042	
Group*distday	IF		2	IF		5	-0.05276	0.1790	18	-0.29	0.7716	
Group*distday	IF		2	IF		6	-0.2724	0.1735	18	-1.57	0.1338	
Group*distday	IF		2	IF		7	-0.1924	0.2158	18	-0.89	0.3842	
Group*distday	IF		2	ad_lib		0	0.1458	0.2821	18	0.52	0.6116	
Group*distday	IF		2	ad_lib		1	0.4299	0.2708	18	1.59	0.1298	
Group*distday	IF		2	ad_lib		2	0.2915	0.2258	18	1.29	0.2132	
Group*distday	IF		2	ad_lib		3	0.1385	0.2577	18	0.54	0.5974	
Group*distday	IF		2	ad_lib		4	-0.00721	0.2742	18	-0.03	0.9793	
Group*distday	IF		2	ad_lib		5	0.4665	0.2687	18	1.74	0.0997	
Group*distday	IF		2	ad_lib		6	-0.05569	0.2546	18	-0.22	0.8293	
Group*distday	IF		2	ad_lib		7	-0.04476	0.2510	18	-0.18	0.8604	
Group*distday	IF		3	IF		4	-0.01313	0.1585	18	-0.08	0.9349	
Group*distday	IF		3	IF		5	-0.1658	0.2052	18	-0.81	0.4295	
Group*distday	IF		3	IF		6	-0.3855	0.1498	18	-2.57	0.0192	
Group*distday	IF		3	IF		7	-0.3055	0.2071	18	-1.48	0.1574	
Group*distday	IF		3	ad_lib		0	0.03270	0.3056	18	0.11	0.9160	
Group*distday	IF		3	ad_lib		1	0.3168	0.2954	18	1.07	0.2978	
Group*distday	IF		3	ad_lib		2	0.1784	0.2540	18	0.70	0.4915	
Group*distday	IF		3	ad_lib		3	0.02547	0.2839	18	0.09	0.9295	
Group*distday	IF		3	ad_lib		4	-0.1203	0.2990	18	-0.40	0.6922	
Group*distday	IF		3	ad_lib		5	0.3534	0.2931	18	1.21	0.2435	
Group*distday	IF		3	ad_lib		6	-0.1688	0.2809	18	-0.60	0.5555	
Group*distday	IF		3	ad_lib		7	-0.1578	0.2774	18	-0.57	0.5764	
Group*distday	IF		4	IF		5	-0.1527	0.2446	18	-0.62	0.5403	
Group*distday	IF		4	IF		6	-0.3723	0.1702	18	-2.19	0.0421	
Group*distday	IF		4	IF		7	-0.2924	0.2542	18	-1.15	0.2651	
Group*distday	IF		4	ad_lib		0	0.04583	0.3120	18	0.15	0.8849	
Group*distday	IF		4	ad_lib		1	0.3299	0.3028	18	1.09	0.2902	
Group*distday	IF		4	ad_lib		2	0.1915	0.2617	18	0.73	0.4737	
Group*distday	IF		4	ad_lib		3	0.03860	0.2908	18	0.13	0.8959	
Group*distday	IF		4	ad_lib		4	-0.1071	0.3072	18	-0.35	0.7313	
Group*distday	IF		4	ad_lib		5	0.3665	0.2999	18	1.22	0.2374	
Group*distday	IF		4	ad_lib		6	-0.1556	0.2884	18	-0.54	0.5961	
Group*distday	IF		4	ad_lib		7	-0.1447	0.2846	18	-0.51	0.6173	
Group*distday	IF		5	IF		6	-0.2196	0.1855	18	-1.18	0.2519	
Group*distday	IF		5	IF		7	-0.1397	0.1782	18	-0.78	0.4433	
Group*distday	IF		5	ad_lib		0	0.1985	0.3065	18	0.65	0.5253	
Group*distday	IF		5	ad_lib		1	0.4826	0.2960	18	1.63	0.1204	
Group*distday	IF		5	ad_lib		2	0.3442	0.2554	18	1.35	0.1944	
Group*distday	IF		5	ad_lib		3	0.1913	0.2842	18	0.67	0.5095	
Group*distday	IF		5	ad_lib		4	0.04555	0.2994	18	0.15	0.8808	
Group*distday	IF		5	ad_lib		5	0.5192	0.2973	18	1.75	0.0978	
Group*distday	IF		5	ad_lib		6	-0.00293	0.2827	18	-0.01	0.9918	
Group*distday	IF		5	ad_lib		7	0.007992	0.2803	18	0.03	0.9776	
Group*distday	IF		6	IF		7	0.07994	0.1919	18	0.42	0.6819	
Group*distday	IF		6	ad_lib		0	0.4181	0.2955	18	1.42	0.1741	
Group*distday	IF		6	ad_lib		1	0.7023	0.2857	18	2.46	0.0244	
Group*distday	IF		6	ad_lib		2	0.5638	0.2426	18	2.32	0.0320	
Group*distday	IF		6	ad_lib		3	0.4109	0.2735	18	1.50	0.1503	
Group*distday	IF		6	ad_lib		4	0.2652	0.2897	18	0.92	0.3721	
Group*distday	IF		6	ad_lib		5	0.7389	0.2845	18	2.60	0.0182	
Group*distday	IF		6	ad_lib		6	0.2167	0.2725	18	0.80	0.4368	
Group*distday	IF		6	ad_lib		7	0.2276	0.2676	18	0.85	0.4062	
Group*distday	IF		7	ad_lib		0	0.3382	0.2928	18	1.16	0.2632	
Group*distday	IF		7	ad_lib		1	0.6223	0.2829	18	2.20	0.0411	
Group*distday	IF		7	ad_lib		2	0.4839	0.2402	18	2.01	0.0591	
Group*distday	IF		7	ad_lib		3	0.3310	0.2711	18	1.22	0.2379	
Group*distday	IF		7	ad_lib		4	0.1852	0.2871	18	0.65	0.5269	
Group*distday	IF		7	ad_lib		5	0.6589	0.2834	18	2.33	0.0320	
Group*distday	IF		7	ad_lib		6	0.1368	0.2689	18	0.51	0.6172	
Group*distday	IF		7	ad_lib		7	0.1477	0.2675	18	0.55	0.5877	
Group*distday	ad_lib		0	ad_lib		1	0.2841	0.2608	18	1.09	0.2903	
Group*distday	ad_lib		0	ad_lib		2	0.1457	0.2130	18	0.68	0.5027	
Group*distday	ad_lib		0	ad_lib		3	-0.00723	0.2158	18	-0.03	0.9736	
Group*distday	ad_lib		0	ad_lib		4	-0.1530	0.2525	18	-0.61	0.5523	
Group*distday	ad_lib		0	ad_lib		5	0.3207	0.3066	18	1.05	0.3094	
Group*distday	ad_lib		0	ad_lib		6	-0.2015	0.2344	18	-0.86	0.4013	
Group*distday	ad_lib		0	ad_lib		7	-0.1905	0.2769	18	-0.69	0.5002	
Group*distday	ad_lib		1	ad_lib		2	-0.1384	0.2189	18	-0.63	0.5351	
Group*distday	ad_lib		1	ad_lib		3	-0.2913	0.2226	18	-1.31	0.2070	
Group*distday	ad_lib		1	ad_lib		4	-0.4371	0.1685	18	-2.59	0.0183	
Group*distday	ad_lib		1	ad_lib		5	0.03661	0.2928	18	0.13	0.9019	
Group*distday	ad_lib		1	ad_lib		6	-0.4856	0.2386	18	-2.04	0.0568	
Group*distday	ad_lib		1	ad_lib		7	-0.4746	0.3341	18	-1.42	0.1725	
Group*distday	ad_lib		2	ad_lib		3	-0.1529	0.1413	18	-1.08	0.2935	
Group*distday	ad_lib		2	ad_lib		4	-0.2987	0.2130	18	-1.40	0.1778	
Group*distday	ad_lib		2	ad_lib		5	0.1750	0.2077	18	0.84	0.4104	
Group*distday	ad_lib		2	ad_lib		6	-0.3471	0.1968	18	-1.76	0.0947	
Group*distday	ad_lib		2	ad_lib		7	-0.3362	0.2389	18	-1.41	0.1763	
Group*distday	ad_lib		3	ad_lib		4	-0.1457	0.1757	18	-0.83	0.4177	
Group*distday	ad_lib		3	ad_lib		5	0.3279	0.2344	18	1.40	0.1788	
Group*distday	ad_lib		3	ad_lib		6	-0.1942	0.1697	18	-1.14	0.2673	
Group*distday	ad_lib		3	ad_lib		7	-0.1833	0.2289	18	-0.80	0.4337	
Group*distday	ad_lib		4	ad_lib		5	0.4737	0.2793	18	1.70	0.1071	
Group*distday	ad_lib		4	ad_lib		6	-0.04848	0.1940	18	-0.25	0.8055	
Group*distday	ad_lib		4	ad_lib		7	-0.03756	0.2805	18	-0.13	0.8950	
Group*distday	ad_lib		5	ad_lib		6	-0.5222	0.2103	18	-2.48	0.0231	
Group*distday	ad_lib		5	ad_lib		7	-0.5112	0.1937	18	-2.64	0.0167	
Group*distday	ad_lib		6	ad_lib		7	0.01093	0.2180	18	0.05	0.9606	
Diet2*distday		HFD	0		HFD	1	0.2688	0.2857	18	0.94	0.3592	
Diet2*distday		HFD	0		HFD	2	-0.1102	0.2450	18	-0.45	0.6582	
Diet2*distday		HFD	0		HFD	3	-0.1073	0.2463	18	-0.44	0.6681	
Diet2*distday		HFD	0		HFD	4	-0.07170	0.2698	18	-0.27	0.7935	
Diet2*distday		HFD	0		HFD	5	-0.1846	0.3203	18	-0.58	0.5715	
Diet2*distday		HFD	0		HFD	6	-0.5796	0.2429	18	-2.39	0.0282	
Diet2*distday		HFD	0		HFD	7	-0.5007	0.2747	18	-1.82	0.0850	
Diet2*distday		HFD	0		SD	0	0.01161	0.3350	18	0.03	0.9727	
Diet2*distday		HFD	0		SD	1	0.01061	0.3277	18	0.03	0.9745	
Diet2*distday		HFD	0		SD	2	-0.3213	0.2941	18	-1.09	0.2891	
Diet2*distday		HFD	0		SD	3	-0.3640	0.3178	18	-1.15	0.2670	
Diet2*distday		HFD	0		SD	4	-0.5585	0.3305	18	-1.69	0.1082	
Diet2*distday		HFD	0		SD	5	-0.1246	0.3262	18	-0.38	0.7069	
Diet2*distday		HFD	0		SD	6	-0.4715	0.3154	18	-1.49	0.1523	
Diet2*distday		HFD	0		SD	7	-0.4595	0.3126	18	-1.47	0.1589	
Diet2*distday		HFD	1		HFD	2	-0.3790	0.2133	18	-1.78	0.0925	
Diet2*distday		HFD	1		HFD	3	-0.3761	0.2209	18	-1.70	0.1059	
Diet2*distday		HFD	1		HFD	4	-0.3405	0.1564	18	-2.18	0.0430	
Diet2*distday		HFD	1		HFD	5	-0.4534	0.2747	18	-1.65	0.1162	
Diet2*distday		HFD	1		HFD	6	-0.8484	0.2269	18	-3.74	0.0015	
Diet2*distday		HFD	1		HFD	7	-0.7695	0.3217	18	-2.39	0.0279	
Diet2*distday		HFD	1		SD	0	-0.2572	0.3128	18	-0.82	0.4217	
Diet2*distday		HFD	1		SD	1	-0.2582	0.3048	18	-0.85	0.4081	
Diet2*distday		HFD	1		SD	2	-0.5901	0.2671	18	-2.21	0.0403	
Diet2*distday		HFD	1		SD	3	-0.6328	0.2931	18	-2.16	0.0446	
Diet2*distday		HFD	1		SD	4	-0.8273	0.3074	18	-2.69	0.0149	
Diet2*distday		HFD	1		SD	5	-0.3934	0.3021	18	-1.30	0.2092	
Diet2*distday		HFD	1		SD	6	-0.7403	0.2909	18	-2.54	0.0203	
Diet2*distday		HFD	1		SD	7	-0.7282	0.2876	18	-2.53	0.0209	
Diet2*distday		HFD	2		HFD	3	0.002909	0.1322	18	0.02	0.9827	
Diet2*distday		HFD	2		HFD	4	0.03854	0.1967	18	0.20	0.8469	
Diet2*distday		HFD	2		HFD	5	-0.07435	0.1859	18	-0.40	0.6938	
Diet2*distday		HFD	2		HFD	6	-0.4693	0.1801	18	-2.61	0.0179	
Diet2*distday		HFD	2		HFD	7	-0.3905	0.2241	18	-1.74	0.0985	
Diet2*distday		HFD	2		SD	0	0.1218	0.2779	18	0.44	0.6663	
Diet2*distday		HFD	2		SD	1	0.1208	0.2677	18	0.45	0.6571	
Diet2*distday		HFD	2		SD	2	-0.2110	0.2258	18	-0.93	0.3623	
Diet2*distday		HFD	2		SD	3	-0.2538	0.2555	18	-0.99	0.3337	
Diet2*distday		HFD	2		SD	4	-0.4483	0.2710	18	-1.65	0.1155	
Diet2*distday		HFD	2		SD	5	-0.01441	0.2662	18	-0.05	0.9574	
Diet2*distday		HFD	2		SD	6	-0.3612	0.2528	18	-1.43	0.1702	
Diet2*distday		HFD	2		SD	7	-0.3492	0.2498	18	-1.40	0.1791	
Diet2*distday		HFD	3		HFD	4	0.03563	0.1646	18	0.22	0.8311	
Diet2*distday		HFD	3		HFD	5	-0.07726	0.2130	18	-0.36	0.7211	
Diet2*distday		HFD	3		HFD	6	-0.4722	0.1556	18	-3.04	0.0071	
Diet2*distday		HFD	3		HFD	7	-0.3934	0.2151	18	-1.83	0.0840	
Diet2*distday		HFD	3		SD	0	0.1189	0.3035	18	0.39	0.6998	
Diet2*distday		HFD	3		SD	1	0.1179	0.2943	18	0.40	0.6934	
Diet2*distday		HFD	3		SD	2	-0.2139	0.2562	18	-0.83	0.4147	
Diet2*distday		HFD	3		SD	3	-0.2567	0.2836	18	-0.91	0.3773	
Diet2*distday		HFD	3		SD	4	-0.4512	0.2977	18	-1.52	0.1469	
Diet2*distday		HFD	3		SD	5	-0.01732	0.2926	18	-0.06	0.9535	
Diet2*distday		HFD	3		SD	6	-0.3641	0.2810	18	-1.30	0.2114	
Diet2*distday		HFD	3		SD	7	-0.3521	0.2781	18	-1.27	0.2216	
Diet2*distday		HFD	4		HFD	5	-0.1129	0.2540	18	-0.44	0.6620	
Diet2*distday		HFD	4		HFD	6	-0.5079	0.1767	18	-2.87	0.0101	
Diet2*distday		HFD	4		HFD	7	-0.4290	0.2640	18	-1.63	0.1215	
Diet2*distday		HFD	4		SD	0	0.08331	0.3105	18	0.27	0.7915	
Diet2*distday		HFD	4		SD	1	0.08230	0.3021	18	0.27	0.7884	
Diet2*distday		HFD	4		SD	2	-0.2496	0.2644	18	-0.94	0.3577	
Diet2*distday		HFD	4		SD	3	-0.2923	0.2910	18	-1.00	0.3284	
Diet2*distday		HFD	4		SD	4	-0.4868	0.3059	18	-1.59	0.1289	
Diet2*distday		HFD	4		SD	5	-0.05295	0.2999	18	-0.18	0.8618	
Diet2*distday		HFD	4		SD	6	-0.3998	0.2889	18	-1.38	0.1833	
Diet2*distday		HFD	4		SD	7	-0.3878	0.2858	18	-1.36	0.1916	
Diet2*distday		HFD	5		HFD	6	-0.3950	0.1927	18	-2.05	0.0552	
Diet2*distday		HFD	5		HFD	7	-0.3161	0.1851	18	-1.71	0.1048	
Diet2*distday		HFD	5		SD	0	0.1962	0.3045	18	0.64	0.5275	
Diet2*distday		HFD	5		SD	1	0.1952	0.2953	18	0.66	0.5169	
Diet2*distday		HFD	5		SD	2	-0.1367	0.2577	18	-0.53	0.6024	
Diet2*distday		HFD	5		SD	3	-0.1794	0.2843	18	-0.63	0.5358	
Diet2*distday		HFD	5		SD	4	-0.3739	0.2984	18	-1.25	0.2262	
Diet2*distday		HFD	5		SD	5	0.05994	0.2960	18	0.20	0.8418	
Diet2*distday		HFD	5		SD	6	-0.2869	0.2827	18	-1.01	0.3237	
Diet2*distday		HFD	5		SD	7	-0.2749	0.2807	18	-0.98	0.3404	
Diet2*distday		HFD	6		HFD	7	0.07885	0.1992	18	0.40	0.6970	
Diet2*distday		HFD	6		SD	0	0.5912	0.2929	18	2.02	0.0587	
Diet2*distday		HFD	6		SD	1	0.5902	0.2838	18	2.08	0.0521	
Diet2*distday		HFD	6		SD	2	0.2583	0.2439	18	1.06	0.3036	
Diet2*distday		HFD	6		SD	3	0.2155	0.2723	18	0.79	0.4390	
Diet2*distday		HFD	6		SD	4	0.02103	0.2874	18	0.07	0.9425	
Diet2*distday		HFD	6		SD	5	0.4549	0.2827	18	1.61	0.1250	
Diet2*distday		HFD	6		SD	6	0.1081	0.2711	18	0.40	0.6948	
Diet2*distday		HFD	6		SD	7	0.1201	0.2673	18	0.45	0.6586	
Diet2*distday		HFD	7		SD	0	0.5123	0.2905	18	1.76	0.0948	
Diet2*distday		HFD	7		SD	1	0.5113	0.2810	18	1.82	0.0855	
Diet2*distday		HFD	7		SD	2	0.1795	0.2415	18	0.74	0.4669	
Diet2*distday		HFD	7		SD	3	0.1367	0.2700	18	0.51	0.6188	
Diet2*distday		HFD	7		SD	4	-0.05782	0.2848	18	-0.20	0.8414	
Diet2*distday		HFD	7		SD	5	0.3761	0.2813	18	1.34	0.1978	
Diet2*distday		HFD	7		SD	6	0.02925	0.2680	18	0.11	0.9143	
Diet2*distday		HFD	7		SD	7	0.04126	0.2666	18	0.15	0.8787	
Diet2*distday		SD	0		SD	1	-0.00101	0.2509	18	-0.00	0.9968	
Diet2*distday		SD	0		SD	2	-0.3329	0.2049	18	-1.62	0.1216	
Diet2*distday		SD	0		SD	3	-0.3756	0.2076	18	-1.81	0.0871	
Diet2*distday		SD	0		SD	4	-0.5701	0.2430	18	-2.35	0.0306	
Diet2*distday		SD	0		SD	5	-0.1363	0.2951	18	-0.46	0.6498	
Diet2*distday		SD	0		SD	6	-0.4831	0.2256	18	-2.14	0.0461	
Diet2*distday		SD	0		SD	7	-0.4711	0.2666	18	-1.77	0.0942	
Diet2*distday		SD	1		SD	2	-0.3319	0.2107	18	-1.57	0.1327	
Diet2*distday		SD	1		SD	3	-0.3746	0.2142	18	-1.75	0.0974	
Diet2*distday		SD	1		SD	4	-0.5691	0.1622	18	-3.51	0.0025	
Diet2*distday		SD	1		SD	5	-0.1352	0.2819	18	-0.48	0.6371	
Diet2*distday		SD	1		SD	6	-0.4821	0.2297	18	-2.10	0.0502	
Diet2*distday		SD	1		SD	7	-0.4701	0.3216	18	-1.46	0.1611	
Diet2*distday		SD	2		SD	3	-0.04277	0.1361	18	-0.31	0.7569	
Diet2*distday		SD	2		SD	4	-0.2373	0.2051	18	-1.16	0.2624	
Diet2*distday		SD	2		SD	5	0.1966	0.2000	18	0.98	0.3386	
Diet2*distday		SD	2		SD	6	-0.1502	0.1895	18	-0.79	0.4383	
Diet2*distday		SD	2		SD	7	-0.1382	0.2300	18	-0.60	0.5554	
Diet2*distday		SD	3		SD	4	-0.1945	0.1692	18	-1.15	0.2653	
Diet2*distday		SD	3		SD	5	0.2394	0.2257	18	1.06	0.3030	
Diet2*distday		SD	3		SD	6	-0.1074	0.1634	18	-0.66	0.5191	
Diet2*distday		SD	3		SD	7	-0.09542	0.2204	18	-0.43	0.6701	
Diet2*distday		SD	4		SD	5	0.4339	0.2690	18	1.61	0.1241	
Diet2*distday		SD	4		SD	6	0.08706	0.1868	18	0.47	0.6468	
Diet2*distday		SD	4		SD	7	0.09908	0.2701	18	0.37	0.7180	
Diet2*distday		SD	5		SD	6	-0.3468	0.2025	18	-1.71	0.1039	
Diet2*distday		SD	5		SD	7	-0.3348	0.1865	18	-1.80	0.0894	
Diet2*distday		SD	6		SD	7	0.01202	0.2099	18	0.06	0.9550	

Observations	87	
Variables	3	


Simple Statistics	
	WkWt	WkGlucose	WkKetone	
Mean	382.3367011	94.12720306	0.8711494253	
StD	38.9002985	8.19821076	0.4549781571	


Correlation Matrix	
	WkWt	WkGlucose	WkKetone	
WkWt	1.0000	0.4797	0.2765	
WkGlucose	0.4797	1.0000	0.1323	
WkKetone	0.2765	0.1323	1.0000	


Eigenvalues of the Correlation Matrix	
	Eigenvalue	Difference	Proportion	Cumulative	
1	1.61714585	0.72983887	0.5390	0.5390	
2	0.88730698	0.39175981	0.2958	0.8348	
3	0.49554717		0.1652	1.0000	


Eigenvectors	
	Prin1	Prin2	Prin3	
WkWt	0.666259	-.129538	-.734383	
WkGlucose	0.609841	-.472123	0.636548	
WkKetone	0.429177	0.871963	0.235559	


Observations	86	
Variables	3	


Simple Statistics	
	WkWt	WkGlucose	WkKetone	
Mean	409.5214424	92.89147287	0.6994186047	
StD	26.6158970	7.72694832	0.5081634089	


Correlation Matrix	
	WkWt	WkGlucose	WkKetone	
WkWt	1.0000	0.0714	-.1779	
WkGlucose	0.0714	1.0000	0.0362	
WkKetone	-.1779	0.0362	1.0000	


Eigenvalues of the Correlation Matrix	
	Eigenvalue	Difference	Proportion	Cumulative	
1	1.18164300	0.15711937	0.3939	0.3939	
2	1.02452363	0.23069027	0.3415	0.7354	
3	0.79383337		0.2646	1.0000	


Eigenvectors	
	Prin1	Prin2	Prin3	
WkWt	0.721128	0.139597	0.678592	
WkGlucose	0.148654	0.925498	-.348362	
WkKetone	-.676665	0.352089	0.646651	
